# Supplementary material for: ‘You’re just there, alone in your room with your thoughts’: a qualitative study about the psychosocial impact of the COVID-19 pandemic among young people living in the UK
Source: BMJ Open. 2022 Feb 8;12(2):e053676. doi: 10.1136/bmjopen-2021-053676 (PMC8829834; doi:10.1136/bmjopen-2021-053676)
Supplement: Author's manuscript [file bmjopen-2021-053676.draft_revisions.pdf]

BMJ Open is committed to open peer review. As part of this commitment we make the peer review history of every article we publish publicly available.

When an article is published we post the peer reviewers' comments and the authors' responses online. We also post the versions of the paper that were used during peer review. These are the versions that the peer review comments apply to.

The versions of the paper that follow are the versions that were submitted during the peer review process. They are not the versions of record or the final published versions. They should not be cited or distributed as the published version of this manuscript.

BMJ Open is an open access journal and the full, final, typeset and author-corrected version of record of the manuscript is available on our site with no access controls, subscription charges or pay-per-view fees (http://bmjopen.bmj.com).

If you have any questions on BMJ Open's open peer review process please email info.bmjopen@bmj.com

BMJ Open

'You're just there, alone in your room with your thoughts:' A qualitative study about the psychosocial impact of lockdown among young people during the COVID-19 pandemic

Journal:	*BMJ Open*
Manuscript ID	bmjopen-2021-053676
Article Type:	Original research
Date Submitted by the Author:	19-May-2021

Complete List of Authors:	McKinlay, Alison; University College London Research Department of Epidemiology and Public Health, Department of Behavioural Science and Health<br>May, Tom; University College London, Behavioural Science and Health, Institute of Epidemiology and Health Care<br>Dawes, Jo; University College London Research Department of Epidemiology and Public Health, Department of Behavioural Science and Health<br>Fancourt, Daisy; University College London Research Department of Epidemiology and Public Health, Department of Behavioural Science and Health<br>Burton, Alexandra; University College London Research Department of Epidemiology and Public Health, Department of Behavioural Science and Health

Keywords:	QUALITATIVE RESEARCH, COVID-19, MENTAL HEALTH, SOCIAL MEDICINE

SCHOLARONE™
Manuscripts

BMJ

I, the Submitting Author has the right to grant and does grant on behalf of all authors of the Work (as defined in the below author licence), an exclusive licence and/or a non-exclusive licence for contributions from authors who are: i) UK Crown employees; ii) where BMJ has agreed a CC-BY licence shall apply, and/or iii) in accordance with the terms applicable for US Federal Government officers or employees acting as part of their official duties; on a worldwide, perpetual, irrevocable, royalty-free basis to BMJ Publishing Group Ltd ("BMJ") its licensees and where the relevant Journal is co-owned by BMJ to the co-owners of the Journal, to publish the Work in this journal and any other BMJ products and to exploit all rights, as set out in our licence.

The Submitting Author accepts and understands that any supply made under these terms is made by BMJ to the Submitting Author unless you are acting as an employee on behalf of your employer or a postgraduate student of an affiliated institution which is paying any applicable article publishing charge ("APC") for Open Access articles. Where the Submitting Author wishes to make the Work available on an Open Access basis (and intends to pay the relevant APC), the terms of reuse of such Open Access shall be governed by a Creative Commons licence – details of these licences and which Creative Commons licence will apply to this Work are set out in our licence referred to above.

Other than as permitted in any relevant BMJ Author's Self Archiving Policies, I confirm this Work has not been accepted for publication elsewhere, is not being considered for publication elsewhere and does not duplicate material already published. I confirm all authors consent to publication of this Work and authorise the granting of this licence.

For peer review only

'You're just there, alone in your room with your thoughts:' A qualitative study about the psychosocial impact of lockdown among young people during the COVID-19 pandemic

Authors:

Alison. R McKinlay, [a] a.mckinlay@ucl.ac.uk

Tom May, [a] t.may@ucl.ac.uk

Jo Dawes, [a] joanna.dawes@ucl.ac.uk

Daisy Fancourt, [a] d.fancourt@ucl.ac.uk

& Alexandra Burton [a] a.burton@ucl.ac.uk

[a] *Institute of Epidemiology & Health Care, University College London, London, United Kingdom*

Corresponding author: Alison McKinlay, PhD, CPsychol, University College London, Department of Behavioural Science and Health, Institute of Epidemiology and Health Care, 1-19 Torrington Place, London, WC1E 7HB.

Declaration of interest

None

Funding

The Covid-19 Social Study was funded by the Nuffield Foundation [WEL/FR-000022583], but the views expressed are those of the authors and not necessarily the Foundation. The study was also supported by the MARCH Mental Health Network funded by the Cross-Disciplinary Mental Health Network Plus initiative supported by UK Research and Innovation [ES/S002588/1], and by the Wellcome Trust [221400/Z/20/Z]. DF was funded by the Wellcome Trust [205407/Z/16/Z].

Acknowledgements

The researchers are grateful for the support of the British Youth Music Theatre during recruitment. Many thanks to Louise Baxter, Rana Conway and Sara Esser for their help with conducting interviews. Thank you to Katey Warren and Henry Aughterson who provided feedback on the themes and subthemes.

Author Contribution Statement

DF conceived the study and DF and AB designed the study. AB, AM, JD and TM collected data for the study, analysed and interpreted the data. AM wrote the first draft with input from AB, JD and TM. All authors provided critical revisions, read, and approved the submitted manuscript. All authors had full access to the data in the study and can take responsibility for the integrity and accuracy of the data.

Data Availability Statement

The data are not publicly available due to their containing information that could compromise the privacy of research participants.

For peer review only

Abstract

Background: Adolescents and young adults have been greatly affected by quarantine measures during the COVID-19 pandemic, but little is understood about how restrictions have affected their wellbeing and social life.

Objectives: We aimed to learn more about how UK quarantine measures affected the social lives, mental health, and wellbeing of adolescents and young adults.

Design: Qualitative interview study.

Setting: Data collection took place remotely across the UK via audio or video call, between June 2020 and January 2021.

Participants: We conducted semi-structured interviews with 37 participants (13-24 years) to elicit their views.

Results: Four themes were generated during analysis: a) concerns about disruption to education, b) missing social contact, c) changes to social relationships, d) improved wellbeing during lockdown. Although participants reported an increased awareness of mental health and stronger relationship ties, many said they struggled with a decline in mental health, lack of support, and concern about socialising after the pandemic.

Conclusions: Findings suggest that some young people felt less stigma talking about their mental health now compared to pre-pandemic, in part facilitated by initiatives through schools, universities, and employers. However, many are worried about how the pandemic has affected their education and social connections and may require additional support.

Keywords – Qualitative Research, Adolescence, Young Adults, COVID-19, Mental Health, Social Support

Article Summary

- We interviewed a wide range of adolescents and young adults from across the UK about their experiences during the COVID-19 pandemic.
- Data collection took place during and in-between several national "lockdown" periods and we elicited their views about the different stages of the pandemic.
- We did not ask participants to provide information about sociodemographic status and it is possible our sample is biased towards those who had the digital and economic means to participate in a remote interview.

For peer review only

Introduction

When coronavirus disease 2019 (COVID-19) was formally declared a pandemic (World Health Organisation, 2020) many governments imposed social distancing restrictions to suppress the virus, including self-isolation and mobility constraints. Whilst these measures are perceived to have been necessary to minimise contact and virus transmission, there is evidence to suggest that they have resulted in adverse psychological consequences across general populations (Niedzwiedz et al., 2020; Pierce et al., 2020).

Lessons learned after previous epidemics suggest that youth experience measures aimed at preventing the spread of viruses as particularly distressing and traumatic (Sprang & Silman, 2013) with the potential for some effects to be long-lasting, even after restrictions are lifted (Brooks et al., 2020; Kwong et al., 2020). Although youth are less likely to experience severe symptomology or hospitalisation with COVID-19 (Bhopal et al., 2021; Ludvigsson, 2020), the impact of quarantine represents a significant mental health threat (Fegert et al., 2020). Emerging evidence suggests that young people have experienced the greatest decline in mental health in the first wave of COVID-19, in comparison to people in other age ranges (Fancourt et al., 2020).

Reasons for this reported decline in mental health among younger people are likely to be multifaceted. Firstly, school closures and education disruptions remove routine, structure and opportunities for socialisation (Lee, 2020), which may increase loneliness and isolation. For children or young people in need of mental health support, closing down schools and universities removed access to coping resources or infrastructures located in these settings (e.g. mental health services and peer support). Secondly, the age span from adolescence to young adulthood is a particularly sensitive one as young people experience major life transitions. Disruption to these transitions can cause uncertainty and anxiety, as documented in young adults experiencing job insecurity resultant from the pandemic (Ganson et al., 2021) or students experiencing the cancellation of their studies (Lee, 2020). Finally, beyond these individual factors, some adolescents and young adults faced additional stressors during COVID-19, including a decline in parental wellbeing, child maltreatment, and bereavement, all of which may compound any wider changes in circumstances initiated by the pandemic (e.g. online learning, home schooling) (Romanou & Belton, 2020; Young Minds, 2021). These experiences were likely felt most acutely by those already affected by socioeconomic marginalisation and deprivation (Waite et al., 2020).

In these contexts, young people and adolescents were at risk of unique psychosocial consequences from the pandemic compared to other population groups, and while others have sought to quantify the impact of the pandemic on young people's mental health, there is limited work exploring young

For peer review only - http://bmjopen.bmj.com/site/about/guidelines.xhtml

people's individual perceptions. In this research, we sought to explore adolescent and young adult experiences during the COVID-19 pandemic in the UK, specifically to understand how and why social distancing restrictions affected social lives, mental health, and wellbeing.

Method

We undertook a qualitative interview study to explore the experiences of adolescents (aged 13-17) and young adults (aged 18-24) throughout the COVID-19 pandemic in the UK. This work forms part of the ongoing COVID-19 Social Study (CSS), which is a national panel survey established in March 2020, where researchers have been investigating the psychosocial wellbeing of people in the UK throughout the pandemic (i.e., Bu, Steptoe, Mak, et al., 2020). The research was reviewed and given approval by the University College London Ethics Committee (London, Project ID: 14895/005).

Participant recruitment

We used a combination of convenience and purposive sampling strategies. Eligibility criteria included: aged 13-24, living in the UK, able to speak English in order to read the study information and to understand and sign a study consent form. All participants aged 16-24 provided written informed consent. Participants aged 13-15 provided assent and a parent provided written informed consent. Those who returned a signed informed consent form also completed a demographics questionnaire (See Table 1 for participant demographics).

Participants were recruited via the CSS study newsletter, through partner organisations working with young people, personal contacts and for young adults aged 18+, via social media. No participant response rates were recorded. All interested individuals were invited to contact the study team for further information. In considering the evidence that certain groups have faced unique challenges during the COVID-19 pandemic (Fancourt et al. 2020), we screened those who registered their interest to ensure participants were from a range of diverse backgrounds in terms of age, gender, ethnicity, education level, and living situation. No participants were previously known to the interviewers prior to recruitment. Potential participants received a Study Information Sheet and all were given the opportunity to ask questions. All participants who participated in interviews were emailed an online £10 shopping voucher as a token of appreciation for their time.

Data collection

Research interviews were the only source of data collected during the study. In light of the quarantine measures in place throughout data collection, all participant interviews took place remotely, either over telephone or video call. Experienced, postgraduate, male and female, qualitative health researchers conducted all interviews (AB, AM, JD, RC, SE, TM, LB). All interviewers have previously

For peer review only

carried out interviews about mental health and social life throughout the COVID-19 pandemic for other CSS research work (Aughterson et al., 2021; Burton et al., 2020; May et al., 2021).

We conducted data collection as two separate groups, to ensure any newly generated themes discussed by participants in each age group (as identified by the researcher) were sufficiently explored during data collection and analysis. Interviews followed a semi-structured format (range: 17-65 minutes, average 38). For adolescents aged 13-17, interviews ranged from 17 to 46 minutes (average: 33 minutes) and for young adults aged 18-24 from 29 to 65 minutes (average: 44 minutes). As the study was designed to elicit experiences of social isolation and social restrictions on mental health, wellbeing and social lives, topic guide questions were informed by social integration and sense of coherence theories (Antonovsky, 1979; Berkman, Glass, Brissette, & Seeman, 2005). The interview topic guide included questions on participants' lives before the pandemic, understanding of and adherence to government restrictions, mental health, and thoughts about the future (for question examples, see Figure 1).

Figure 1 Interview Guide Examples

- *How do you feel about the changes that have been brought about by Covid-19?*
- *Have they had any impact on your mental health or wellbeing?*
- *What are the things most bothering you at the moment?*
- *Has the pandemic meant that you have any worries for the future?*

Data analysis

We audio recorded interviews, which were transcribed verbatim by a transcription service. Returned transcripts were double checked for anonymity and accuracy, then imported into NVivo version 12. We did not use member checking methods during the study. Interrogation of the interview data was informed by phenomenology, with a focus on each participant's own experience. We incorporated reflexive thematic analysis (Braun & Clarke, 2019, 2020), and used a combination of inductive and deductive coding approaches. First, an initial coding framework was developed based on the theoretical concepts explored in the interview topic guide. This was updated with new codes that were grounded in the data identified while reading through the transcripts. AM and TM then carried out line-by-line coding of interview transcripts and created the deductive coding framework. AM, TM and JD double coded three transcripts per group (adolescents, young adults) to ensure consistency in coding topics of salience. The list of themes and subthemes were developed iteratively: AM presented themes and subthemes during weekly internal meetings with the CSS research team, in order to gain

For peer review only - http://bmjopen.bmj.com/site/about/guidelines.xhtml

formative feedback on the comprehensibility of codes and preliminary findings. Findings were revised based on this feedback.

Patient and public involvement

Patients or members of the public were not involved with the design, conduct, reporting or dissemination of this research.

Results

We recruited 37 participants between June 2020 and January 2021. Participant characteristics are described in Table 1. The average age of participants was 18 (range 13-24 years old). The majority were female (62%) and White British (73%). All participants lived with others (e.g., housemates, family, caregiver, spouse). Most participants were in secondary school or university (84%) and living at home with their parents (78%). Sixteen participants reported that they had an existing physical (7) and/or mental health condition (16).

Table 1 Self-reported demographic characteristics

	Participants n = 37
Age	
13-14	4
15-16	12
17-18	8
19-20	2
21-22	6
23-24	5
Sex	
Female	23
Male	14
Ethnicity	
White British	27
Mixed Race*	5
Asian and Asian British**	4
Qualifications	
No qualifications	9
GSCE	9
A Levels	13
Undergraduate	5
Postgraduate	1
Employment	
Still at school	17
At university	14
Fulltime employment	3
Apprenticeship	1

For peer review only - http://bmjopen.bmj.com/site/about/guidelines.xhtml

Self-employed	1
Unemployed	1

* Mixed Race – White and Black African, White and Black Caribbean, White and North African, Other Mixed Background. **Asian or Asian British - Indian, Punjabi, Pakistani, Malaysian.

We identified four overarching themes about the impact of the pandemic on mental health and wellbeing: a) Concerns about disruption to education, b) Missing social contact during lockdown, c) Changes to social relationships, and d) Improved wellbeing during lockdown. Themes and subthemes are illustrated in Figure 2.

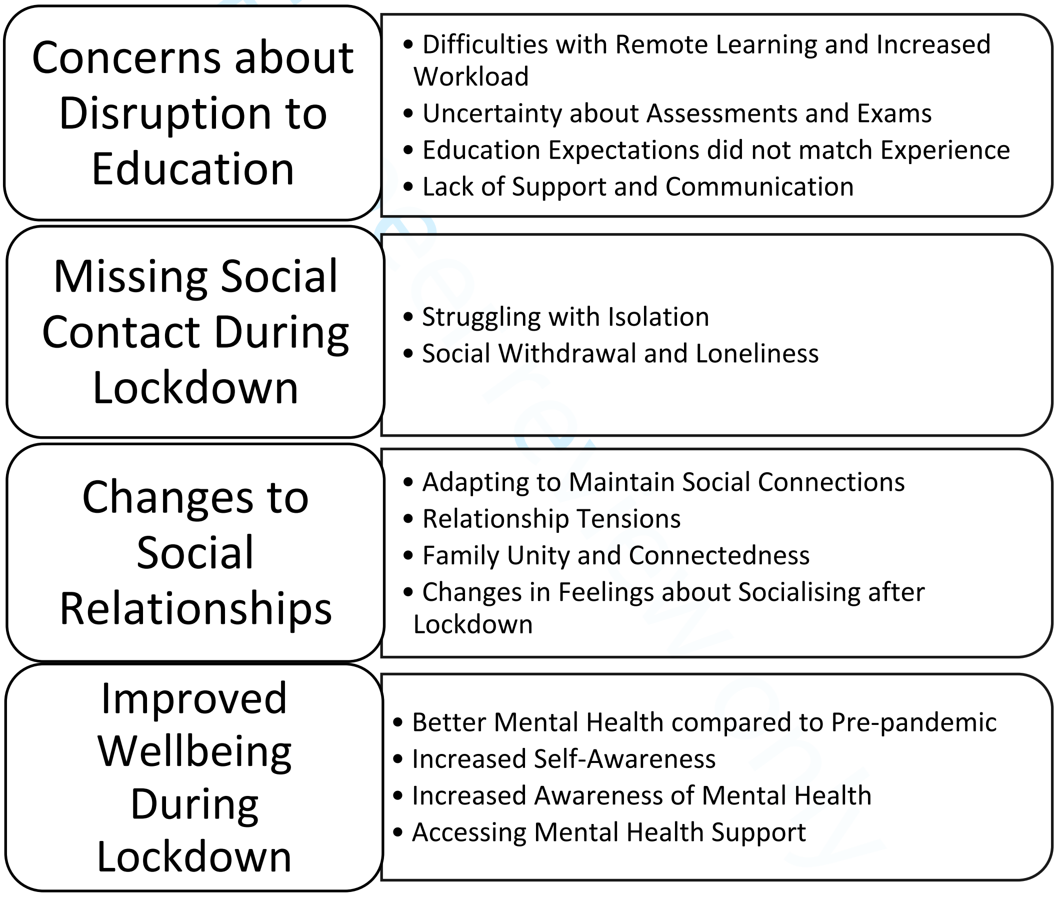

Figure 2 Themes and sub-themes

Theme One: Concerns about Disruption to Education

The cancellation of assessments and closure of schools and universities were described as a source of loss, concern, and uncertainty for most participants. Many felt unsupported in their transition to online, remote learning and others worried about the longer-term impact on their future prospects.

For peer review only - http://bmjopen.bmj.com/site/about/guidelines.xhtml

Difficulties with Remote Learning and Increased Workload

Inability to concentrate, feelings of boredom and a lack of stimulation created difficulties with remote learning for some participants.

> *"I was just staring at a take home exam paper with my brain just completely blue screened. You know, just completely not functioning. I think that just general stimulus vacuum of lockdown massively contributed to that because that's an experience I've never had before." P56, male, aged 18-22*

For those living at home with their families, having to share a workspace with multiple siblings at once, or feeling confined to a bedroom to study because everyone in the household was working from home, meant that "*days felt like night.*"

> *"When I've sat in my room all day, there's no distinguishing between the room where I sleep and the room where I do all my work." P20, male, aged 13-17*

After several months of social distancing and online learning, some participants felt that the move to remote online learning had increased their workload compared to face-to-face learning.

> *"My school…said we'll do live lessons, but then a teacher, instead of doing live lessons, just put the PowerPoint online, and then we just had to take notes from that. And they'd say, make four pages of notes on this, do the questions and then that's your work done for the lesson, which wasn't very good, because instead of spending one hour a lesson, I'd spend two or three hours." P68, male, aged 13-17*

The impact of this increased workload on future exams and ultimately grades created an additional source of concern and uncertainty among participants.

> *"I know that the virus has definitely had a big toll on how much I'm thinking about [exams], because there's so much uncertainty… My worries about exams [are] more like: 'how am I going to get the content done in time,' not how am I going to be graded." P49, female, aged 13-17*

Uncertainty about Assessments and Exams

Participants discussed feeling stressed, particularly at the start of lockdown, when there was a lack of clear guidance over how schools and universities would manage coursework and exams amid closures.

> *"University was probably biggest stress at the start because there was an uncertain period when we first went into lockdown, where all the universities were rushing to work out what*

For peer review only

they were going to do. At this point I was looking at handing in a dissertation and sitting three or four exams, and I didn't know if it was still happening." P54, female, aged 18-22

When announcements were made that exams would be cancelled, many said that they felt upset or disappointed and reported feeling robbed of the chance to finish their education as planned.

"I'm actually really upset that I couldn't sit my exams, so yes. It was a bit of an anticlimax, because I kind of wanted all of the build-up and the apprehension, to finalise, what, two years' worth of… Well, my entire education built up to this. And when it was halted… I found it a bit difficult." p59, female, aged 18-22

Some participants reported that they had studied hard outside of school hours to improve their grades even before the pandemic hit and were anxious that this additional effort would not be taken into account when considering their grade predictions.

"Not doing exams, I was gutted. I can't prove myself and I'm being given a letter that they think I could get... they didn't see how hard I was working…they don't see how hard we work outside of school… It's just annoying that we can't prove ourselves." P34, female, aged 13-17

Education Expectations did not match Experience

Many participants spoke of feeling disappointment that their social plans for secondary school or university had not materialised due to closures and disruptions. Symbolic events tied to the start or end of education (such as "Freshers week", "Leavers assembly" or prom) were cancelled and described as another source of loss during the pandemic.

"We didn't get the finale of Year 13 like we normally do where there's like a prom and things like that and it was all just a bit rushed so we didn't get to say bye." P53, male, aged 18-22

Some said that they felt they had "missed out" on an experience that they saw as a rite of passage and one that they had been looking forward to.

"Because we were planning on going out so much and that all being cancelled, it was awful. The day when I was meant to have prom I put my prom dress on just because I wanted to… it was like the idea of it all being cancelled I felt like I was missing out." P55, female, aged 13-17

Lack of Support and Communication

Many participants described feeling that there was a lack of support for their mental health and wellbeing from education providers during the pandemic.

For peer review only

"The school hasn't been great in terms of providing help… the general attitude is: 'it's okay not to be okay, but we're not going to do anything about it.' I know a couple of other schools have a similar situation where you feel like, do they know that you're struggling, but they're not going to do anything." P61, female, aged 13-17

In cases where support was offered, some said that they had experienced this as performative rather than genuine.

"And I feel really let down by the school…Our head of year did a wellbeing check and it didn't even seem genuine, it just seemed like she had to do it for her ego or something like that..." P34, female, aged 13-17

Others described poorly managed communication and a lack of information from education providers about pandemic-related changes, which left students uncertain about their options for rearranging assessments and coursework.

"It would have been nice if I could speak to my personal tutor or something about it, but I think everyone was so busy at the beginning trying to record lectures and put them online, and everything was a bit hectic. So, if there was support it wasn't very clear or easy to find," P64, female, aged 18-22

Poor communication and a perceived lack of support from education providers also resulted in some students feeling forgotten about or unfairly treated. This led to feelings of anger, frustration and a sense that their experiences were not valued.

"At the beginning, kind of March to May period, the response was absolutely shambolic and pretty malevolent to be honest…telling people to go home at their own expense up to and including sort of international students being told that they had to leave within a week even if that meant that their flights were two grand or something." P56, male, aged 18-22

Theme Two: Missing Social Contact During Lockdown

Many participants reported a decline in wellbeing due to feeling lonely and isolated, particularly at the start of lockdown. Being unable to see friends or family and feeling that they were missing out on important life events was a source of sadness and frustration. Some withdrew themselves even further from online social activities after government restrictions were imposed.

Struggling with Isolation

For peer review only - http://bmjopen.bmj.com/site/about/guidelines.xhtml

Most participants said that their mental health was affected by social distancing restrictions. Many described this as being due to feeling socially restricted or "trapped" at home and being unable to maintain their usual activities and relationships.

> *"I'm not a very anxious person. I've struggled with mental health anyway, but… I think in the beginning of lockdown, being trapped at home wasn't ideal in terms of my mental health." P59, female, aged 18-22*

The feeling of being inside and at home for an indeterminate period of time was overwhelming for some, leading to a desire to overcompensate for what was perceived as "lost time" when restrictions began to ease after the first wave of the virus.

> *"As soon as restrictions were relaxed enough that you could meet someone socially distanced, I was down at my friends' houses that are 100m down the road, three or four times a week, just to get out of my house. I got so frustrated being cooped up." P55, female, aged 13-17*

The social distancing restrictions were especially challenging for participants who had prior mental health concerns, many of whom reported that their wellbeing had declined.

> *"…during lockdown, I got horrendously depressed. So, I've had stuff going on mentally, but I was finding it tough. The social isolation made everything crash really badly." P39, male, aged 18-22*

Social Withdrawal and Loneliness

While many participants had adapted their lives in response to social distancing restrictions, some, particularly adolescents, described socialising less during the lockdown and reported feelings of loneliness or social withdrawal.

> *"I don't really speak to much of my mates as I used to. It's made me quite distanced with quite a lot of people." P66, female, aged 13-17*

At the start of the lockdown, some participants felt uncertain or overwhelmed by the move to online social interactions and made a conscious decision to withdraw from this method of contact.

> *"Before lockdown, I never used video calls or phoning people anyway. I spoke to people through messaging a lot, but I never used Zoom. I never heard of it to be honest. So, when I was suddenly forced to do that, it was just too much, so I've stopped talking to people." P20, male, aged 13-17*

For peer review only - http://bmjopen.bmj.com/site/about/guidelines.xhtml

Boredom was commonly reported by a majority of participants and lowered their motivation to interact with others. Some participants said that they had nothing to talk about with their peers, or that friends were uninterested in keeping in contact. Therefore, efforts to maintain social connection ceased and usual social ties disrupted.

> *"I definitely tried to keep in touch with my school friends more at the start, but then it felt very one-sided, so I stopped making all the effort and I haven't spoken to a few of them in a few months." P44, female, aged 13-17*

Participants described feeling lonely throughout different stages of the pandemic, as though the world felt smaller from being at home during the lockdown without any social contact.

> *"There was some socialising over Zoom but overall, I think my world just sort of contracted fairly significantly…" P56, male, aged 18-22*

Several participants felt lonely even in the presence of others as restrictions began to ease, with many associating their feelings of loneliness with a lack of physical contact and closeness.

> *"So, even though we are back at school, we're still struggling through the same things. We're still feeling lonely, that we're not able to have that physical contact, which I'm looking forward to having if the pandemic gets better." P51, female, aged 13-17*

Theme Three: Changes to Social Relationships

Relationship Tensions

Participants spoke of stress, anxiety and anger regarding family and friendship tensions during the pandemic. For some, this was due to not being permitted to see their friends, which led to relationship breakdowns.

> *"What's changed is it's put a lot of pressure on relationships, like with my boyfriend and a lot of people I know of have broken up with their boyfriends or girlfriends because of this quarantine and not being able to see them every day, or be around them as much." P19, female, aged 13-17*

For others, differences in opinions between friends led to arguments over what was acceptable or not when following the social distancing rules.

> *"One of my mates, my mate, I actually ended up having an argument, because she was, like, oh, you've got to come and see me." P66, female, aged 13-17*

For peer review only - http://bmjopen.bmj.com/site/about/guidelines.xhtml

Participants living with family members said that they experienced difficulties getting along with parents and siblings during lockdown, particularly when living together in small homes or when everyone was working from home.

> *"I don't always get on with my family very well and we would be in each other's faces a hell of a lot and that got very difficult at times… I'd say it gets worse when we have to spend more time together." P38, male, aged 18-22*

> *"I think everyone just got a little bit stir crazy seeing the same walls and the same people." P44, female, aged 13-17*

Relationship Unity and Connectedness

Despite relationship tensions, participants also described feeling settled and closer with their family members during the pandemic and lockdown periods.

> *"My relationship with my parents is great and it's improved. It was at a great point this year and I think better than it's ever been. And I think lockdown was a good time just to spend more time with them." P37, female, aged 20-24*

Many were reminded about the importance of family and were resolute to make more effort to connect with others in the future.

> *"I won't take for granted as much, like just standing outside talking to my neighbour, phone calls with my [relative]. Just the little things like that. Because obviously it's so easy in normal life just to brush them aside and say, 'oh, I'll ring my [relative] later.' So now I take the time to ring my [relative] and talk to her." P33, female, aged 13-17*

Some participants had difficult conversations during the lockdown that they otherwise might not have had, for instance, what they would do if a family member became ill with the virus. Having frank and open conversations helped some to feel more connected than before the pandemic.

> *"I definitely think [the pandemic] forced us to talk about things that we wouldn't always want to talk about. We had to discuss as a family what would happen to my dad if he was to get ill…We discussed those things already in general because we are quite open about that but I think it increased the intensity and frequency of those conversations about death and what would happen and health and things like that… it's helped us to be more open with each other" P41, female, aged 20-24*

Adapting to Maintain Social Connections

For peer review only - http://bmjopen.bmj.com/site/about/guidelines.xhtml

In response to social distancing measures, participants described the ways in which they had adapted their social lives, including trying new methods of online communication.

> *"The idea of not knowing when you're next going to be in a crowded place, really able one on one to chat to people I think has just made people search for other ways of connecting with people and that's online." P41, female, aged 20-24*

Some said they were using dating apps, chatting through gaming platforms and sharing COVID-related memes as a way "to cope with difficult things" and enhance their sense of connectedness with others. New friendships were formed during lockdown with the help of group chats on gaming and social media platforms (i.e., "Whatsapp").

> *"I have made a bunch of friends that are from online around my age from playing a game...so during the pandemic instead of chatting with my friends I'd be playing a game with them almost every day. Well, every day for a few hours chatting, having fun which was nice." P21, male, aged 13-17*

Changes in Feelings about Socialising after Lockdown

Around half of participants described how their feelings about in-person social interactions had changed since the start of the pandemic. Some described this in terms of feeling more vigilant about being around other people and of the risks associated with future virus transmission.

> *"I think I'm going to be a lot more cautious about other people, obviously, like people you don't know being quite close to you, physically." P45, male, aged 13-17*

Although individual concern about their own risk of catching COVID was not widespread in the group overall, some participants still reported feeling safer during lockdown away from other people and less exposed to the perceived risk of catching COVID-19.

> *"I felt less anxious about everything when we were in full lockdown and that the lifting out of lockdown, that's quite scary in many ways for many different people…obviously introducing more risk into your life, whereas when everyone was in full lockdown, I feel like there was something quite comforting in a weird way about that." P37, female, aged 20-24*

Adolescents in particular said they felt apprehensive about future social situations from not having been exposed to "*real life*" and becoming accustomed to life in lockdown.

> *"I just feel like I've lost the ability to form coherent sentences. I know that sounds really stupid. It's just been really hard." P34, female, aged 13-17*

For peer review only - http://bmjopen.bmj.com/site/about/guidelines.xhtml

Anxiety about socialising was especially present in those returning to school after months in lockdown, following difficulties in keeping in touch with friends and maintaining positive friendships.

> *"I was nervous, because a lot changed during quarantine. I think people got really tetchy staying at home, so there were a lot of arguments. Loads of people fell out of contact with one another…So, it was harder for us to find common things to talk about, we were just speaking about the same topics again and again. So, going back [to school, I] was really nervous because we didn't know what we would talk about." P49, female, aged 13-17*

Theme Four: Improved Wellbeing During Lockdown

For those who described busy and stressful lives prior to the pandemic, social distancing restrictions provided a chance to pause and reflect, with resultant mental health benefits. Having more choice about how to spend free time was experienced by participants as relaxing and removed previous sources of stress. For some, their wellbeing was reportedly enhanced by support from others and an increased public awareness of mental health self-management during lockdown.

Improved Mental Health Compared to Pre-pandemic

In comparison to a majority of participants who reported struggling with their wellbeing during the pandemic, a smaller group of adolescents described how their mental health had improved during the first lockdown.

> *"Well, before lockdown, my mental health wasn't great… my sleep schedule was all over the place, school stress, but actually, lockdown gave me a chance to focus and make myself a lot better in terms of the positive emotions" P49, female, aged 13-17*

They described busy or stressful school lives prior to the pandemic. School closures and a shift to online learning, particularly for those who were not preparing for their exams, meant that some had less work to do. The lockdown therefore provided an opportunity to relax and reflect.

> *"I think it's been just I guess inconvenience rather than a full crisis in my eyes. It probably should have affected me more but, yes, I quite like it in quarantine to be honest. I don't have to get up, I can just chill around. It's a lot nicer than having to rush around constantly." P21, male, aged 13-17*

As previously reported, the closure of schools and university meant an increased workload for some, but the flexibility of choosing what to do between lessons and during free time meant the lockdown felt "*like a really long summer holiday*" for others.

For peer review only - http://bmjopen.bmj.com/site/about/guidelines.xhtml

"I was happy when school got cancelled… it was still hard to do the work at home, but it was just because it was just like, in between I could just do whatever I wanted, so it was just better, sometimes I just go and play in the garden, football, or just be on my phone or the PS4." p28, male, aged 13-17

Increased Self-Awareness

Throughout the course of the pandemic, some participants had more time to take up hobbies and exercise, but several young adults also commented on the personal growth and increased self-awareness that they had observed.

"[This experience has] taught me to take everything a day as it comes. And then see what happens. But I won't be surprised if, in a good way, that things change and it happens for the better….it allowed me to challenge myself more... now things have started to flow better with how I am as a person." P60, female, aged 18-22

Some were reflecting on the stark changes in their lives during the pandemic compared with pre-pandemic and said they felt differently about how they wanted their life to be when social distancing restrictions eased.

"First, I think someone hit the brakes, I think, like everything stopped…[Life is] no longer that constant, hectic mess of going out…overall, it's a lot less intense maybe, now." P39, male, 18-22

Increased Awareness of Mental Health

Some participants said they felt an increased awareness of their own mental health and how to protect it during the pandemic, through initiatives driven by workplaces and education providers.

"My organisation did allocate three weeks of wellbeing leave which is a really interesting move. And that was on top of annual leave. And that was complementary leave because the reason why they did it is that they said that it's a really hard time and they just think everyone needs the break." P37, female, aged 20-24

Some said they had not paid attention to their mental health in the past compared to now.

"[the pandemic has] shown that health and mental health and mental state is important. As it always was, but I don't think I realised it as much as I would now." P46, male, aged 13-17

For peer review only - http://bmjopen.bmj.com/site/about/guidelines.xhtml

This perceived change in mental health awareness helped make it feel easier to talk to others about their wellbeing, and for some, this "opened up" a dialogue with friends and family that had never been achieved before.

> *"It opened up really big conversations within our family about mental health that I don't think they've ever had before like the generations. So those are two really big positives for me I think. Both myself and opening that up wider...It was just conversations that we'd never had before." P37, female, aged 20-24*

Accessing Mental Health Support

Some participants sought support to help cope with pandemic-related distress, either informally through friends and family, or formally through education providers, health services, or employers.

> *"I did talk to one of my teachers actually at school, on Teams…And she helped me and a lot of friends about things to do during lockdown." P50, female, aged 13-17*

In some cases, regular offers of support and check-ins by educational staff were sufficiently reassuring to know that help was on hand if needed.

> *"[University staff would] email every week, checking in. They gave me two… deadline extensions, for my work, which helped me a lot. And my tutor was really helpful. He did check in every couple of weeks to make sure I was all right. Even over the summer, when they haven't necessarily been at uni. So, that was good." P57, female, aged 18-22*

Discussion

We sought the views of adolescents and young adults about their wellbeing and social lives during the COVID-19 pandemic using detailed qualitative interviews. Young people described mental health consequences associated with education closures and lockdown measures, including loneliness, frustration, a sense of loss about cancelled plans and assessments, and uncertainty about socialising in the future. Our findings highlight how some felt paradoxically isolated but also hesitant about reintegrating with others as the lockdown restrictions ease. Despite the challenges, we also found evidence of a potentially positive impact of the pandemic, including an increased awareness of mental health self-management, and having more open conversations about mental health compared to pre-pandemic.

While the long-term effect of school closures on young people's health, income and productivity is yet to be fully realised (Viner et al., 2020), disruptions to education on this scale have not occurred since the second World War (d'Orville, 2020). These changes will likely have long term consequences

For peer review only - http://bmjopen.bmj.com/site/about/guidelines.xhtml

requiring ongoing support (The British Academy, 2021), with particular attention needed on how sociodemographic factors have affected young peoples' experiences during the pandemic (Scott et al., 2021). Around 60% of students living in affluent areas had access to online learning during the pandemic, compared with 23% of students living in more deprived areas (Cullinane & Montacute, 2020). A loss of learning has already been observed by teaching staff in the UK (Kim et al., 2021), and our findings suggest this may be compounded by increased workloads, sharing small workspaces at home, and difficulties with concentration. If unaddressed, this may restrict opportunities for employment and social mobility available to young people in the future (The British Academy, 2021). It is essential that those without the resources to work optimally from home are supported, to ensure that educational and social inequalities are not exacerbated, both as COVID-19 persists and in other future scenarios where remote learning may be required.

It is estimated more than half of students have experienced adverse psychological wellbeing during the COVID-19 pandemic (Office for National Statistics, 2020). We found that paradoxically, while some participants felt isolated and lonely, they were also apprehensive about social distancing restrictions easing and life returning to "normal". Graded or phased approaches for those returning to education (Viner et al., 2021) and employment are recommended as lockdown restrictions ease (Habersaat et al., 2020). Academic challenges have been frequently reported by young people during the pandemic, particularly among high-school aged youth (Scott et al., 2021). We also noted concern among those who had access to online learning but were unable to sit exams and finish their coursework. Several young people felt they did not demonstrate their full potential when their exams were cancelled and did not want to be known as the generation who were given a "free pass". Teaching staff may be well-placed to address these concerns by clearly communicating revised assessment plans and providing reassurance about the assessment process for future students affected by pandemic-related education disruptions. Teachers also require clear guidance and coordination from government and educational authorities to be able to deliver these messages (Kim et al., 2021), which were absent during the first wave of educational establishment closures.

Young people have reported some of the highest rates of loneliness during the pandemic (Bu et al., 2020), which have been associated with a decline in psychological wellbeing (Lee et al., 2020). Initiatives that enable young people to easily connect with others and access support for their mental health while adhering to social distancing guidelines are greatly needed (Lee et al., 2020). Participants in our study frequently cited mental health consequences due to social isolation and loneliness. These findings build on those shown in previous research involving young people during the first national lockdown (i.e., Demkowicz et al., 2020), including the sense of loss and uncertainty frequently reported by this group. Our results build on this by suggesting how social isolation led to these effects,

For peer review only - http://bmjopen.bmj.com/site/about/guidelines.xhtml

including what felt like missed opportunities to create memories with friends and missed social events seen as rites of passage in young adulthood.

The effects of isolation and loneliness for young people are likely to persist for some time, even after social distancing restrictions have lifted (Loades et al., 2020). The path ahead will require additional resources for education providers and coordinated efforts from policy makers, education authorities, and health and social care to provide reassurance that service provision is responsive to the needs of young people (Kim & Asbury, 2020). This response should be formulated in collaboration with young people to ensure that any future support incorporates appropriate coping strategies and protective activities (Efuribe et al., 2020), such as those identified by our study, including check-ins from education providers and wellbeing initiatives from employers. These responses could help to validate the unique struggles that many young people have faced throughout the different stages of the pandemic (Efuribe et al., 2020).

Despite the many negative effects of the pandemic on mental health in young people identified to date, evidence also suggests that adolescents' prosocial behaviours have increased during the pandemic, including providing practical support to others and donating to charities in need (Alvis et al., 2020). It is encouraging that some participants in this study reported feeling more able to talk about their mental health compared with pre-pandemic. This was, at times, facilitated by top-down efforts from education providers, employers, and parents. In this study and others (Aughterson et al., 2021), we found that many have been reminded of the importance of family and friends during adversity. These changes represent a potentially positive side effect of the pandemic, providing they are supported and maintained.

Strengths and Limitations

To our knowledge, this is the first UK study to use qualitative interviewing to learn more about the experiences of adolescents and young people during the COVID-19 pandemic. Purposive recruitment methods increased the variability of experiences within the group of participants interviewed. Those whom we spoke with were a range of ages, had different living situations and health statuses. Data were gathered throughout several key time points of the UK pandemic response, including as schools were reopening after the first lockdown, and as the second wave of the virus had begun. We relied on participants self-identifying to take part in the research, which may have meant that the sample whose experiences were more positive than those less comfortable or with less time to take part in research. Remote interviewing techniques may have excluded some groups with limited online access; however, we also offered to conduct telephone calls and remote data collection methods enabled us to speak to people from across the UK.

For peer review only - http://bmjopen.bmj.com/site/about/guidelines.xhtml

Conclusions

When the pandemic began and restrictions were introduced, many young people lost a sense of certainty, community and belonging provided by their routines, social circles, and education providers. Our findings highlight how support for adolescents and young people to develop and maintain their social connections and access mental health support is needed even after the virus is controlled. Most participants in our study struggled with their wellbeing and loneliness during the pandemic, and our results highlight the vital role of education providers giving regular guidance and support. We also highlight the detrimental impact that an absence of information and support had on mental health. More must be done to ensure offers of support are genuine and reinforced with signposting to information, advice and services when young people do reach out for help.

For peer review only

References

Alvis, L., Douglas, R., Shook, N., & Oosterhoff, B. (2020). Associations between Adolescents' Prosocial Experiences and Mental Health During the COVID-19 Pandemic. *PsyArXiv*. https://psyarxiv.com/2s73n

Antonovsky, A. (1979). *Health, stress, and coping*. Jossey-Bass.

Aughterson, H., McKinlay, A. R., Fancourt, D., & Burton, A. (2021). Psychosocial impact on frontline health and social care professionals in the UK during the COVID-19 pandemic: A qualitative interview study. *BMJ Open*, *11*(2), e047353. https://doi.org/10.1136/bmjopen-2020-047353

Berkman LF, Glass T, Brissette I, Seeman TE. From social integration to health: Durkheim in the new millennium. Social Science & Medicine. 2000;51(6):843-57.

Bhopal, S. S., Bagaria, J., Olabi, B., & Bhopal, R. (2021). Children and young people remain at low risk of COVID-19 mortality. *The Lancet. Child & Adolescent Health*. https://doi.org/10.1016/S2352-4642(21)00066-3

Braun, V., & Clarke, V. (2019). Reflecting on reflexive thematic analysis. *Qualitative Research in Sport, Exercise and Health*, *11*(4), 589–597. https://doi.org/10.1080/2159676X.2019.1628806

Braun, V., & Clarke, V. (2020). One size fits all? What counts as quality practice in (reflexive) thematic analysis? *Qualitative Research in Psychology*, 1–25. https://doi.org/10.1080/14780887.2020.1769238

For peer review only - http://bmjopen.bmj.com/site/about/guidelines.xhtml

For peer review only

Brooks, S. K., Webster, R. K., Smith, L. E., Woodland, L., Wessely, S., Greenberg, N., & Rubin, G. J. (2020). The psychological impact of quarantine and how to reduce it: Rapid review of the evidence. *The Lancet*, *395*(10227), 912–920. https://doi.org/10.1016/S0140-6736(20)30460-8

Bu, F., Steptoe, A., & Fancourt, D. (2020). Loneliness during a strict lockdown: Trajectories and predictors during the COVID-19 pandemic in 38,217 United Kingdom adults. *Social Science & Medicine*, 113521. https://doi.org/10.1016/j.socscimed.2020.113521

Burton, A., McKinlay, A. R., Aughterson, H., & Fancourt, D. (2020). Impact of the Covid-19 pandemic on the mental health and wellbeing of adults with mental health conditions in the UK: A qualitative interview study. *Medrxiv: The Preprint Server for Health Sciences*. https://doi.org/10.1101/2020.12.01.20241067

Cullinane, C., & Montacute, R. (2020). *COVID-19 and Social Mobility Impact Brief #1: School Closures*. https://www.suttontrust.com/wp-content/uploads/2021/01/School-Shutdown-Covid-19.pdf

d'Orville, H. (2020). COVID-19 causes unprecedented educational disruption: Is there a road towards a new normal? *PROSPECTS*, *49*(1–2), 11–15. https://doi.org/10.1007/s11125-020-09475-0

Demkowicz, O., Ashworth, E., O'Neill, A., Hanley, T., & Pert, K. (2020). *Teenagers' Experiences of Life in Lockdown: The TELL study briefing*. https://www.seed.manchester.ac.uk/education/research/impact/teenagers-experiences-of-life-in-lockdown/

Efuribe, C., Barre-Hemingway, M., Vaghefi, E., & Suleiman, A. B. (2020). Coping With the COVID-19 Crisis: A Call for Youth Engagement and the Inclusion of Young People in Matters That Affect Their Lives. *Journal of Adolescent Health*, *67*(1), 16–17. https://doi.org/10.1016/j.jadohealth.2020.04.009

Fancourt, D., Steptoe, A., & Bu, F. (2020). Trajectories of anxiety and depressive symptoms during enforced isolation due to COVID-19 in England: A longitudinal observational study. *The Lancet Psychiatry*. https://doi.org/10.1016/S2215-0366(20)30482-X

For peer review only

For peer review only - http://bmjopen.bmj.com/site/about/guidelines.xhtml

Fegert, J. M., Vitiello, B., Plener, P. L., & Clemens, V. (2020). Challenges and burden of the Coronavirus 2019 (COVID-19) pandemic for child and adolescent mental health: A narrative review to highlight clinical and research needs in the acute phase and the long return to normality. *Child and Adolescent Psychiatry and Mental Health*, *14*(1), 20. https://doi.org/10.1186/s13034-020-00329-3

Ganson, K. T., Tsai, A. C., Weiser, S. D., Benabou, S. E., & Nagata, J. M. (2021). Job Insecurity and Symptoms of Anxiety and Depression Among U.S. Young Adults During COVID-19. *Journal of Adolescent Health*, *68*(1), 53–56. https://doi.org/10.1016/j.jadohealth.2020.10.008

Habersaat, K. B., Betsch, C., Danchin, M., Sunstein, C. R., Böhm, R., Falk, A., Brewer, N. T., Omer, S. B., Scherzer, M., Sah, S., Fischer, E. F., Scheel, A. E., Fancourt, D., Kitayama, S., Dubé, E., Leask, J., Dutta, M., MacDonald, N. E., Temkina, A., … Butler, R. (2020). Ten considerations for effectively managing the COVID-19 transition. *Nature Human Behaviour*, *4*(7), 677–687. https://doi.org/10.1038/s41562-020-0906-x

Kim, L. E., & Asbury, K. (2020). 'Like a rug had been pulled from under you': The impact of COVID-19 on teachers in England during the first six weeks of the UK lockdown. *British Journal of Educational Psychology*, *90*(4), 1062–1083. https://doi.org/10.1111/bjep.12381

Kim, L., Leary, R., & Asbury, K. (2021). 'We need clear directions, if we're going to move forward. It's as simple as that': Teachers' narratives during partial school reopenings in the COVID-19 pandemic. *PsyArXiv*. https://doi.org/10.31234/osf.io/m8scj

Kwong, A. S. F., Pearson, R. M., Smith, D., Northstone, K., Lawlor, D. A., & Timpson, N. J. (2020). Longitudinal evidence for persistent anxiety in young adults through COVID-19 restrictions. *Wellcome Open Research*, *5*, 195. https://doi.org/10.12688/wellcomeopenres.16206.1

Lee, C., Cadigan, C., & Rhew, I. (2020). Increases in Loneliness Among Young Adults During the COVID-19 Pandemic and Association With Increases in Mental Health Problems. *Journal of Adolescent Health*. https://doi.org/10.1016/j.jadohealth.2020.08.009

For peer review only

For peer review only - http://bmjopen.bmj.com/site/about/guidelines.xhtml

Lee, J. (2020). Mental health effects of school closures during COVID-19. *The Lancet Child & Adolescent Health*, *4*(6), 421. https://doi.org/10.1016/S2352-4642(20)30109-7

Loades, M. E., Chatburn, E., Higson-Sweeney, N., Reynolds, S., Shafran, R., Brigden, A., Linney, C., McManus, M. N., Borwick, C., & Crawley, E. (2020). Rapid Systematic Review: The Impact of Social Isolation and Loneliness on the Mental Health of Children and Adolescents in the Context of COVID-19. *Journal of the American Academy of Child & Adolescent Psychiatry*, *59*(11), 1218-1239.e3. https://doi.org/10.1016/j.jaac.2020.05.009

Ludvigsson, J. F. (2020). Systematic review of COVID-19 in children shows milder cases and a better prognosis than adults. *Acta Paediatrica*, *109*(6), 1088–1095. https://doi.org/10.1111/apa.15270

May, T., Aughterson, H., Fancourt, D., & Burton, A. (2021). 'Stressed, uncomfortable, vulnerable, neglected': A qualitative study of the psychological and social impact of the COVID-19 pandemic on UK frontline keyworkers. *SocArXiv*. https://doi.org/10.31235/osf.io/dn43c

Niedzwiedz, C. L., Green, M. J., Benzeval, M., Campbell, D., Craig, P., Demou, E., Leyland, A., Pearce, A., Thomson, R., Whitley, E., & Katikireddi, S. V. (2020). Mental health and health behaviours before and during the initial phase of the COVID-19 lockdown: Longitudinal analyses of the UK Household Longitudinal Study. *Journal of Epidemiology and Community Health*, jech-2020-215060. https://doi.org/10.1136/jech-2020-215060

Office for National Statistics. (2020). *Coronavirus and the impact on students in higher education in England: September to December 2020*. https://www.ons.gov.uk/peoplepopulationandcommunity/educationandchildcare/articles/coronavirusandtheimpactonstudentsinhighereducationinenglandseptembertodecember2020/2020-12-21

Pierce, M., Hope, H., Ford, T., Hatch, S., Hotopf, M., John, A., Kontopantelis, E., Webb, R., Wessely, S., McManus, S., & Abel, K. M. (2020). Mental health before and during the COVID-19

For peer review only - http://bmjopen.bmj.com/site/about/guidelines.xhtml

pandemic: A longitudinal probability sample survey of the UK population. *The Lancet Psychiatry*, *7*(10), 883–892. https://doi.org/10.1016/S2215-0366(20)30308-4

Romanou, E., & Belton, E. (2020). *Isolated and struggling: Social isolation and the risk of child maltreatment, in lockdown and beyond.* https://learning.nspcc.org.uk/media/2246/isolated-and-struggling-social-isolation-risk-child-maltreatment-lockdown-and-beyond.pdf

Scott, S. R., Rivera, K. M., Rushing, E., Manczak, E. M., Rozek, C. S., & Doom, J. R. (2021). "I Hate This": A Qualitative Analysis of Adolescents' Self-Reported Challenges During the COVID-19 Pandemic. *Journal of Adolescent Health*, *68*(2), 262–269. https://doi.org/10.1016/j.jadohealth.2020.11.010

Sprang, G., & Silman, M. (2013). Posttraumatic Stress Disorder in Parents and Youth After Health-Related Disasters. *Disaster Medicine and Public Health Preparedness*, *7*(1), 105–110. https://doi.org/10.1017/dmp.2013.22

The British Academy. (2021). *THE COVID DECADE Understanding the long-term societal impacts of COVID-19*. https://www.thebritishacademy.ac.uk/documents/3238/COVID-decade-understanding-long-term-societal-impacts-COVID-19.pdf

Viner, R. M., Bonell, C., Drake, L., Jourdan, D., Davies, N., Baltag, V., Jerrim, J., Proimos, J., & Darzi, A. (2021). Reopening schools during the COVID-19 pandemic: Governments must balance the uncertainty and risks of reopening schools against the clear harms associated with prolonged closure. *Archives of Disease in Childhood*, *106*(2), 111–113. https://doi.org/10.1136/archdischild-2020-319963

Viner, R. M., Russell, S. J., Croker, H., Packer, J., Ward, J., Stansfield, C., Mytton, O., Bonell, C., & Booy, R. (2020). School closure and management practices during coronavirus outbreaks including COVID-19: A rapid systematic review. *The Lancet Child & Adolescent Health*, *4*(5), 397–404. https://doi.org/10.1016/S2352-4642(20)30095-X

For peer review only - http://bmjopen.bmj.com/site/about/guidelines.xhtml

Waite, P., Pearcey, S., Shum, A., Raw, J., Patalay, P., & Creswell, C. (2020). *How did the mental health of children and adolescents change during early lockdown during the COVID-19 pandemic in the UK?* [Preprint]. PsyArXiv. https://doi.org/10.31234/osf.io/t8rfx

World Health Organisation. (2020). *Coronavirus disease 2019 (COVID-19) Situation Report – 51*. https://www.who.int/docs/default-source/coronaviruse/situation-reports/20200311-sitrep-51-covid-19.pdf?sfvrsn=1ba62e57_10

Young Minds. (2021). *Coronavirus: Impact on young people with mental health needs.* https://youngminds.org.uk/media/4350/coronavirus-report-winter.pdf

For peer review only

Supplementary Material 1: Topic Guide

Please could you describe what normal life was like for you before the pandemic and the social distancing? (can explain before – e.g. staying at home, stopping visiting people's houses, having to stand 2 metres apart, etc)

- Who do you normally live with separated/ extended family?
- Do you have any siblings/ pets?
- School type, do you have friends you would normally visit or would visit you – please tell me about this?
- Do you have any hobbies or things you enjoyed doing out of school time?

UNDERSTANDING AND ADHERENCE TO GUIDELINES

At the moment, are you 'staying at home'?

- Can you tell me what this is like for you and your family?
  - i.e. are you/ your parents going out for shopping or for walk/ exercise
  - Or staying inside with no outside exercise?/ other people dropping off shopping?

- **What do you understand by the 'social distancing' advice that is being given – what does it mean to you? (Ask to list the rules they are aware of)**
- Have you been…
  - Avoiding crowds
  - Keeping distance from others
  - Isolating (staying inside)
  - Avoiding close contact greetings
  - Socialising/going out only with those in your household

Have you been able to stick to the social distancing advice that has been given? Please tell us about why/ why not?

- What helps you to stick to it?
- Or – what are you finding difficult about sticking to the rules?
- What has motivated you to do this?
- Is there anything that makes it easier to do or harder

[COM-B prompts can be used here, to include:]

- Any existing physical or mental health problems
- Group membership/ applicability Whether it feels like the rules apply to you
- Beliefs about whether it will keep you healthy
- Beliefs about whether it will keep other people healthy
- Having to go out for things like food
- Work/ Caring responsibilities, providing emotional support
- Friends suggesting meeting, family or parents wanting to go out
- Government rules/punishments,
- Feelings about losing normal life
- Change of routine/ habits

For peer review only

For peer review only - http://bmjopen.bmj.com/site/about/guidelines.xhtml

SOCIAL LIFE – In terms of before the pandemic, How would you describe your social life before the Covid-19 pandemic started?
Although some of the question seems less directly relevant, we are trying to understand a big picture of how groups affect/ influence how we feel.

- How would you describe the group of people you knew before the pandemic (these may be the same as now)
  - Are they friends or family or both mainly?
  - Do they live near you or far away?
  - How often did you see each other?
- Did you speak to family and friends face to face/online?

- Were you involved in any groups outside school – please describe (examples such as choir, dancing classes, football teams, youth groups/ youth theatre, Scouts/Guides, social activities etc)
- Did you have people who helped you, such as teachers, Scout leaders, parents, grandparents, family friends? How did they help you, for example listening, help with homework, help with mental health?
- Can you tell us about whether or not your friendship groups encouraged you to get involved in things? Did you find you compared your life to theirs?
- Social roles possibly as a young carer/ older sibling

How would you describe your social life now because of Covid-19?

Please tell us about this – make clear that we are talking about after the start of the pandemic, to see if anything has changed.

Prompts include:

- How would you describe the group of people you know now?
  - Are they friends or family or both mainly?
  - Do they live near you or far away?
  - How often did you see each other?
- Do you speak to family and friends face to face/online?
- Are you seeing or speaking to teachers and students as part of school online?

- Are you involved in any online groups outside school – please describe (examples such as online choir, dancing classes, , youth groups/ youth theatre, Scouts/Guides, social activities etc)
- Do you have people who help you, such as teachers, Scout leaders, parents, grandparents, family friends? How did they help you, for example listening, help with homework, help with mental health?
- Can you tell us about any ways your friendship groups influence you such as encouraging you to get involved in things? Do you find you compare your life and theirs?
- Social roles possibly as a young carer

For peer review only - http://bmjopen.bmj.com/site/about/guidelines.xhtml

MENTAL HEALTH

How do you feel about the changes that have been brought about by Covid-19?
Have they had any impact on your mental health or wellbeing? Please tell us about these

- What are the things most bothering you at the moment?
- Have you experienced any impact on positive emotions? (prompts: concentration, being able to do and achieve things, relationships with others, how well you are managing and feelings of control over your situation?)
- Have you experienced any negative feelings? (prompts: such as, not enjoying things as much, anxiety, worry)
- Could you tell us if you have had any physical symptoms that might be due to being stressed or anxious? (prompts: fatigue, sleep problems, pain, illness symptoms, heart racing)

Have you been doing/ planning anything to help with this?

- Connecting with family or friends online
- Online groups?
- Hobbies/ Reading
- Exercise at home/ outside the home
- Volunteering
- Anything else you can tell me about?

Why are you doing/ not doing these things?

- Helpful/ not helpful – please tell us why
- Enjoyable
- Good for mental health/ wellbeing
- Can't get online, not connected, not comfortable, affordability, confidence in using/ skills
- Skills in using the internet/ communication software
- Living arrangements/ Work/ caring demands
- Peer support/ pressure
- Difficulties/ restriction in physical environment

PROSPECTION

Has the pandemic meant that you have any worries for the future?

How are these different from the worries you had before?

- Sense of control/ powerlessness
- Severity of worries / perspective

Will this change the way you live your life in future?

- The way you connect with others
- How you look after yourself
- How you support others
-

Has this changed what things are important to you?

For peer review only - http://bmjopen.bmj.com/site/about/guidelines.xhtml

Standards for Reporting Qualitative Research (SRQR)*

http://www.equator-network.org/reporting-guidelines/srqr/

Title and abstract

	Page/line no(s).
**Title** - Concise description of the nature and topic of the study Identifying the study as qualitative or indicating the approach (e.g., ethnography, grounded theory) or data collection methods (e.g., interview, focus group) is recommended	1, 1-3
**Abstract** - Summary of key elements of the study using the abstract format of the intended publication; typically includes background, purpose, methods, results, and conclusions	2, 1-17

Introduction

**Problem formulation** - Description and significance of the problem/phenomenon studied; review of relevant theory and empirical work; problem statement	3, 16-30
**Purpose or research question** - Purpose of the study and specific objectives or questions	4, 1-3

Methods

**Qualitative approach and research paradigm** - Qualitative approach (e.g., ethnography, grounded theory, case study, phenomenology, narrative research) and guiding theory if appropriate; identifying the research paradigm (e.g., postpositivist, constructivist/ interpretivist) is also recommended; rationale**	4, 5-6<br>5, 18-19
**Researcher characteristics and reflexivity** - Researchers' characteristics that may influence the research, including personal attributes, qualifications/experience, relationship with participants, assumptions, and/or presuppositions; potential or actual interaction between researchers' characteristics and the research questions, approach, methods, results, and/or transferability	4, 28-32
**Context** - Setting/site and salient contextual factors; rationale**	4, 28
**Sampling strategy** - How and why research participants, documents, or events were selected; criteria for deciding when no further sampling was necessary (e.g., sampling saturation); rationale**	4, 18-24
**Ethical issues pertaining to human subjects** - Documentation of approval by an appropriate ethics review board and participant consent, or explanation for lack thereof; other confidentiality and data security issues	4, 9-10<br>4, 14-16
**Data collection methods** - Types of data collected; details of data collection procedures including (as appropriate) start and stop dates of data collection and analysis, iterative process, triangulation of sources/methods, and modification of procedures in response to evolving study findings; rationale**	4, 29 (types of data)<br>5, 3-5 (responding to evolving findings)

For peer review only

For peer review only - http://bmjopen.bmj.com/site/about/guidelines.xhtml

	5, 27 – 6, 2 (iterative theme generation)<br>6, 4 (dates)
**Data collection instruments and technologies** - Description of instruments (e.g., interview guides, questionnaires) and devices (e.g., audio recorders) used for data collection; if/how the instrument(s) changed over the course of the study	Topic Guide in Supplementary Data
**Units of study** - Number and relevant characteristics of participants, documents, or events included in the study; level of participation (could be reported in results)	Table on p6, 11-13
**Data processing** - Methods for processing data prior to and during analysis, including transcription, data entry, data management and security, verification of data integrity, data coding, and anonymization/de-identification of excerpts	5, 17-27
**Data analysis** - Process by which inferences, themes, etc., were identified and developed, including the researchers involved in data analysis; usually references a specific paradigm or approach; rationale**	5, 17-27
**Techniques to enhance trustworthiness** - Techniques to enhance trustworthiness and credibility of data analysis (e.g., member checking, audit trail, triangulation); rationale**	5, 20

Results/findings

**Synthesis and interpretation** - Main findings (e.g., interpretations, inferences, and themes); might include development of a theory or model, or integration with prior research or theory	p6-16
**Links to empirical data** - Evidence (e.g., quotes, field notes, text excerpts, photographs) to substantiate analytic findings	p6-16

Discussion

**Integration with prior work, implications, transferability, and contribution(s) to the field -** Short summary of main findings; explanation of how findings and conclusions connect to, support, elaborate on, or challenge conclusions of earlier scholarship; discussion of scope of application/generalizability; identification of unique contribution(s) to scholarship in a discipline or field	17-19
**Limitations** - Trustworthiness and limitations of findings	19, 12-23

Other

**Conflicts of interest** - Potential sources of influence or perceived influence on study conduct and conclusions; how these were managed	1, 16-17
**Funding** - Sources of funding and other support; role of funders in data collection, interpretation, and reporting	1, 18-23

For peer review only - http://bmjopen.bmj.com/site/about/guidelines.xhtml

*The authors created the SRQR by searching the literature to identify guidelines, reporting standards, and critical appraisal criteria for qualitative research; reviewing the reference lists of retrieved sources; and contacting experts to gain feedback. The SRQR aims to improve the transparency of all aspects of qualitative research by providing clear standards for reporting qualitative research.

**The rationale should briefly discuss the justification for choosing that theory, approach, method, or technique rather than other options available, the assumptions and limitations implicit in those choices, and how those choices influence study conclusions and transferability. As appropriate, the rationale for several items might be discussed together.

Reference:

O'Brien BC, Harris IB, Beckman TJ, Reed DA, Cook DA. **Standards for reporting qualitative research: a synthesis of recommendations.** *Academic Medicine*, Vol. 89, No. 9 / Sept 2014 DOI: 10.1097/ACM.0000000000000388

For peer review only

BMJ Open

'You're just there, alone in your room with your thoughts:' A qualitative study about the psychosocial impact of lockdown among young people during the COVID-19 pandemic

Journal:	*BMJ Open*
Manuscript ID	bmjopen-2021-053676.R1
Article Type:	Original research
Date Submitted by the Author:	24-Oct-2021
Complete List of Authors:	McKinlay, Alison; University College London Research Department of Epidemiology and Public Health, Department of Behavioural Science and Health<br>May, Tom; University College London, Behavioural Science and Health, Institute of Epidemiology and Health Care<br>Dawes, Jo; University College London Research Department of Epidemiology and Public Health, Department of Behavioural Science and Health<br>Fancourt, Daisy; University College London Research Department of Epidemiology and Public Health, Department of Behavioural Science and Health<br>Burton, Alexandra; University College London Research Department of Epidemiology and Public Health, Department of Behavioural Science and Health
**Primary Subject Heading**:	Public health
Secondary Subject Heading:	Mental health
Keywords:	QUALITATIVE RESEARCH, COVID-19, MENTAL HEALTH, SOCIAL MEDICINE

SCHOLARONE™
Manuscripts

I, the Submitting Author has the right to grant and does grant on behalf of all authors of the Work (as defined in the below author licence), an exclusive licence and/or a non-exclusive licence for contributions from authors who are: i) UK Crown employees; ii) where BMJ has agreed a CC-BY licence shall apply, and/or iii) in accordance with the terms applicable for US Federal Government officers or employees acting as part of their official duties; on a worldwide, perpetual, irrevocable, royalty-free basis to BMJ Publishing Group Ltd ("BMJ") its licensees and where the relevant Journal is co-owned by BMJ to the co-owners of the Journal, to publish the Work in this journal and any other BMJ products and to exploit all rights, as set out in our licence.

The Submitting Author accepts and understands that any supply made under these terms is made by BMJ to the Submitting Author unless you are acting as an employee on behalf of your employer or a postgraduate student of an affiliated institution which is paying any applicable article publishing charge ("APC") for Open Access articles. Where the Submitting Author wishes to make the Work available on an Open Access basis (and intends to pay the relevant APC), the terms of reuse of such Open Access shall be governed by a Creative Commons licence – details of these licences and which Creative Commons licence will apply to this Work are set out in our licence referred to above.

Other than as permitted in any relevant BMJ Author's Self Archiving Policies, I confirm this Work has not been accepted for publication elsewhere, is not being considered for publication elsewhere and does not duplicate material already published. I confirm all authors consent to publication of this Work and authorise the granting of this licence.

For peer review only

'You're just there, alone in your room with your thoughts:' A qualitative study about the psychosocial impact of lockdown among young people during the COVID-19 pandemic

Authors:

Alison. R McKinlay, [a] a.mckinlay@ucl.ac.uk

Tom May, [a] t.may@ucl.ac.uk

Jo Dawes, [a] joanna.dawes@ucl.ac.uk

Daisy Fancourt, [a] d.fancourt@ucl.ac.uk

& Alexandra Burton [a] a.burton@ucl.ac.uk

[a] *Institute of Epidemiology & Health Care, University College London, London, United Kingdom*

Corresponding author: Alison McKinlay, PhD, CPsychol, AFBPsS, University College London, Department of Behavioural Science and Health, Institute of Epidemiology and Health Care, 1-19 Torrington Place, London, WC1E 7HB.

Declaration of interest

None

Funding

The Covid-19 Social Study was funded by the Nuffield Foundation [WEL/FR-000022583], but the views expressed are those of the authors and not necessarily the Foundation. The study was also supported by the MARCH Mental Health Network funded by the Cross-Disciplinary Mental Health Network Plus initiative supported by UK Research and Innovation [ES/S002588/1], and by the Wellcome Trust [221400/Z/20/Z]. DF was funded by the Wellcome Trust [205407/Z/16/Z].

Acknowledgements

The researchers are grateful for the support of the British Youth Music Theatre during recruitment. Many thanks to Louise Baxter, Rana Conway and Sara Esser for their help with conducting interviews. Thank you to Katey Warren and Henry Aughterson who provided feedback on the themes and subthemes. Thank you to the peer reviewers who provided critical feedback and suggestions to improve the manuscript.

Author Contribution Statement

DF and AB designed the study. AB, AM, JD and TM collected data for the study, analysed and interpreted the data. AM wrote the first draft with input from AB, JD and TM. All authors provided critical revisions, read, and approved the submitted manuscript. All authors had full access to the data in the study and can take responsibility for the integrity and accuracy of the data.

Ethical Statement

This research was granted ethical approval by the University College London Ethics Committee (London, Project ID: 14895/005). All participants aged 16-24 provided written informed consent to participate. Participants aged 13-15 provided written assent to participant and a parent also provided their verbal and written informed consent.

Data Availability Statement

For peer review only

The data are not publicly available due to their containing information that could compromise the privacy of research participants.

Abstract

Introduction: Adolescents and young adults have been greatly affected by quarantine measures during the COVID-19 pandemic, but little is understood about how restrictions have affected their wellbeing and social life. We aimed to learn more about how UK quarantine measures affected the social lives, mental health, and wellbeing of adolescents and young adults.

Method: We conducted semi-structured interviews with 37 participants (13-24 years) to elicit their views. Data collection took place remotely across the UK via audio or video call, between June 2020 and January 2021. The data were analysed using reflexive thematic analysis, with particular attention paid to contextual factors (such as age, gender, ethnicity and health status) when analysing each individual transcript.

Results: Authors generated four themes during the qualitative analysis: a) concerns about disruption to education, b) missing social contact during lockdown, c) changes to social relationships and d) improved wellbeing during lockdown. Many participants said they struggled with a decline in mental health during the pandemic, lack of support, and concern about socialising after the pandemic. However, some participants described experiences and changes brought on by the pandemic as helpful, including an increased awareness of mental health and feeling more at ease when talking about it, as well as stronger relationship ties with family members.

Conclusions: Findings suggest that young people may have felt more comfortable when talking about their mental health compared to pre-pandemic, in part facilitated by initiatives through schools, universities, and employers. However, many were worried about how the pandemic has affected their education and social connections, and support for young people should be tailored accordingly around some of these concerns.

Keywords – Qualitative Research, Adolescence, Young Adults, COVID-19, Mental Health, Social Support

Article Summary

- We interviewed a wide range of adolescents and young adults from across the UK about their experiences during the COVID-19 pandemic.
- Data collection took place during, and in-between several national "lockdown" periods and we elicited their views about the different stages of the pandemic.
- Participants in the study described a decline in their mental health following education disruptions and introduction of "lockdown" measures, such as loneliness, frustration, and uncertainty about the future.
- Although young people in the study faced challenges, many also said this was a time of connecting with family and becoming more comfortable discussing their mental health with others.
- We did not ask participants to provide information about sociodemographic status and it is possible the sample is biased towards those who had the digital and economic means to participate in a remote interview.

For peer review only - http://bmjopen.bmj.com/site/about/guidelines.xhtml

Introduction

When coronavirus disease 2019 (COVID-19) was formally declared a pandemic [1] many governments imposed social distancing restrictions to suppress the virus, including social isolation and mobility constraints. Whilst these measures were proposed to minimise contact and virus transmission, there is evidence to suggest that they have resulted in adverse psychological consequences across general populations. [2,3]

Lessons learned from previous epidemics suggest that youth experience measures aimed at preventing the spread of viruses as particularly distressing [4] with the potential for some effects to be long-lasting, even after restrictions have lifted. [5,6] The effect of pandemic-related disruptions in routine may be felt more acutely by children and young people with pre-existing conditions, such as autism spectrum disorder. [7] While youth are less likely to experience severe symptomology or hospitalisation with COVID-19, [8,9] the impact of quarantine represents a significant mental health threat. [10] Emerging evidence suggests that young people have experienced the greatest decline in mental health in the first wave of COVID-19, in comparison to people in other age ranges, [11] and young people identifying as Black and Black British have been most affected in the UK compared to other ethnic groups. [12]

Reasons for this reported decline in mental health among younger people are likely to be multifaceted. Firstly, school closures and education disruptions remove routine, structure and opportunities for socialisation, [13] which may increase loneliness and isolation. For children or young people in need of mental health support, the closing down of schools and universities removed access to coping resources or infrastructures located in these settings (e.g., mental health services and peer support). Secondly, the age span from adolescence to young adulthood is a particularly sensitive one as young people experience major life transitions. Disruption to these transitions can cause uncertainty and anxiety, as documented in young adults experiencing job insecurity resultant from the pandemic [14] or students experiencing the cancellation of their studies. [13] Finally, beyond these individual factors, some adolescents and young adults faced additional stressors during COVID-19, including a decline in parental wellbeing, child maltreatment, and bereavement, all of which may compound changes in circumstances brought on by the pandemic (e.g. online learning, home schooling). [15,16] These experiences were likely felt most acutely by those already affected by socioeconomic marginalisation and deprivation. [17]

In these contexts, young people and adolescents were at risk of unique psychosocial consequences from the pandemic compared to other population groups, and while others have sought to quantify the impact of the pandemic on young people's mental health, there is limited work exploring young

For peer review only - http://bmjopen.bmj.com/site/about/guidelines.xhtml

people's individual perceptions at various stages of the pandemic. In this research, we sought to explore adolescent and young adult experiences during the COVID-19 pandemic in the UK, specifically to understand how and why social distancing restrictions affected social lives, mental health, and wellbeing.

Method

We undertook a qualitative interview study to explore the experiences of adolescents (aged 13-17) and young adults (aged 18-24) throughout the COVID-19 pandemic in the UK. This work forms part of the COVID-19 Social Study (CSS), which is a national panel survey established in March 2020, where researchers have been investigating the psychosocial wellbeing of people in the UK throughout the pandemic. [18] The research was reviewed and given approval by the University College London Ethics Committee (London, Project ID: 14895/005).

Participant recruitment

Eligibility criteria included: aged 13-24, living in the UK, able to speak English in order to read the study information and to understand and sign a study consent form. All participants aged 16-24 provided written informed consent. Participants aged 13-15 provided assent and a parent provided written informed consent. All participants completed a demographics questionnaire (See Table 1 for participant demographics). No participants were previously known to the interviewers prior to recruitment. In order to make an informed decision about whether to participate, potential participants received a Study Information Sheet with further details on the study, data protection measures and links to organisations providing mental health support. All were given the opportunity to ask questions and no one who was offered an interview declined to take part. No participant response rates were recorded. All participants who participated in interviews were emailed an online £10 shopping voucher as a token of appreciation for their time.

We recruited a convenience sample informed by purposive and snowballing sampling strategies. Our purposive recruitment approach was aligned with "quota sampling," whereby a minimum number of participants in each group (based on age, ethnicity, living situation) were recruited to ensure we were able to learn about the richness of experience across groups. [19] In considering the evidence that certain groups have faced unique challenges during the COVID-19 pandemic (Fancourt et al. 2020), we screened those who registered their interest in attempt to ensure participants were from a range of diverse backgrounds in terms of age, gender, ethnicity, education level, and living situation. The study was advertised via the CSS study newsletter, through partner organisations working with young people, personal contacts and for young adults aged 18+, via social media. Although we screened

For peer review only - http://bmjopen.bmj.com/site/about/guidelines.xhtml

participants to ensure people from multiple groups were included, the nature of our recruitment process meant our sample was ultimately recruited by convenience. [19]

Data collection

In light of the quarantine measures in place at various points throughout data collection, all participant interviews took place remotely, either over telephone or video call. Experienced, postgraduate, male and female, qualitative health researchers conducted all interviews (AB, AM, JD, RC, SE, TM, LB). All interviewers have previously carried out interviews about mental health and social life throughout the COVID-19 pandemic for other CSS research work. [20–22] Interviews followed a semi-structured format (range: 17-65 minutes, average 38), with prompts used to focus on each participant's own lived experience of the COVID-19 pandemic. For adolescents aged 13-17, interviews ranged from 17 to 46 minutes (average: 33 minutes) and for young adults aged 18-24 from 29 to 65 minutes (average: 44 minutes). As the study was designed to elicit experiences of social isolation and social restrictions on mental health, wellbeing and social lives, topic guide questions were informed by social integration and sense of coherence theories. [23,24] The interview topic guide included questions on participants' lives before the pandemic, understanding of and adherence to government restrictions, mental health, and thoughts about the future (for question examples, see Figure 1. For full list, see Supplementary file).

[figure 1 about here]

In order to capture some of the nuanced differences between experiences of adolescents compared with young adults, we made several key choices during data collection and analysis. We conducted data collection as two separate groups according to participant age (group 1: adolescents aged 13-17 years, group 2: young adults aged 18-24). We made this decision in order to focus on experiences that were specific to different stages of development in adolescence and "emerging" adulthood. [25] Where possible in our results, efforts have been made to clarify any similarities and differences in participant responses based on their age group. Rather than progressing in a linear fashion from stage to stage during qualitative data analysis, [26] we made a decision to begin coding before data collection had finished. This approach allowed us to reflect on the way questions were asked during interviews and use prompts to learn more about topics of relevance to the research aims.

Data analysis

Research interviews were the only source of data collected and analysed during the study. We audio recorded interviews, which were transcribed verbatim by a transcription service. Transcripts were double checked for anonymity and accuracy, then imported into NVivo version 12. We did not use

For peer review only - http://bmjopen.bmj.com/site/about/guidelines.xhtml

member checking methods during the study. We incorporated reflexive thematic analysis, [27,28] and used a combination of inductive and deductive coding approaches. First, an initial coding framework was developed based on the theoretical concepts explored in the interview topic guide. This was updated with new codes that were grounded in the data identified whilst reading through the transcripts. AM and TM double coded three interview transcripts in the young adult group and discussed these to ensure coder consistency. AM and JD double coded three transcripts in the adolescent group to ensure consistency across coders in identifying topics of salience. AM and TM then carried out line-by-line coding of interview transcripts and created the coding framework. The list of themes and subthemes were developed iteratively: AM presented themes and subthemes during weekly internal meetings with the CSS research team, in order to gain formative feedback on the comprehensibility of codes and preliminary findings. Findings were revised and finalised based on this feedback.

Patient and public involvement

Patients or members of the public were not involved with the design, conduct, reporting or dissemination of this research.

Results

We recruited 37 participants between June 2020 and January 2021. Participant characteristics are described in Table 1. The average age of participants was 18 (range 13-24 years old). The majority were female (62%) and White British (73%). All participants lived with others (e.g., housemates, family, caregiver, spouse). Most participants were in secondary school or university (84%) and living at home with their parents (78%). Sixteen participants reported that they had an existing physical (7) and/or mental health condition (16).

Table 1 Self-reported demographic characteristics

	Participants n = 37
Age	
13-14	4
15-16	12
17-18	8
19-20	2
21-22	6
23-24	5
Sex	
Female	23
Male	14

For peer review only - http://bmjopen.bmj.com/site/about/guidelines.xhtml

Ethnicity	
White British	27
Mixed Race*	5
Asian and Asian British**	4
**Qualifications**	
No qualifications	9
GSCE	9
A Levels	13
Undergraduate	5
Postgraduate	1
**Employment**	
Still at school	17
At university	14
Fulltime employment	3
Apprenticeship	1
Self-employed	1
Unemployed	1

* Participants who identified as Mixed Race also specified that they identify as White and Black African, White and Black Caribbean, White and North African, Other Mixed Background. ** Participants who identified as Asian or Asian British also specified that they identify as Indian, Punjabi, Pakistani, and Malaysian.

We generated four overarching themes about the impact of the pandemic on mental health and wellbeing: a) Concerns about disruption to education, b) Missing social contact during lockdown, c) Changes to social relationships, and d) Improved wellbeing during lockdown. Themes and subthemes are illustrated in Figure 2.

[Figure 2 about here]

Theme One: Concerns about Disruption to Education

The cancellation of assessments and closure of schools and universities were described as a source of loss, concern, and uncertainty for most participants. Many felt unsupported in their transition to online, remote learning and others worried about the longer-term impact of education closures on their future career and learning prospects.

Difficulties with Remote Learning and Increased Workload

Inability to concentrate, feelings of boredom and a lack of stimulation created difficulties with remote learning for some participants.

> *"I was just staring at a take home exam paper with my brain just completely blue screened. You know, just completely not functioning. I think that just general stimulus vacuum of lockdown massively contributed to that because that's an experience I've never had before." P56, male, aged 18-22*

For peer review only - http://bmjopen.bmj.com/site/about/guidelines.xhtml

For those living at home with their families, having to share a workspace with multiple siblings at once, or feeling confined to a bedroom to study because everyone in the household was working from home, meant that "*days felt like night.*"

> *"When I've sat in my room all day, there's no distinguishing between the room where I sleep and the room where I do all my work." P20, male, aged 13-17*

After several months of social distancing and online learning, some participants felt that the move to remote online learning had increased not only their screentime but their workload as well, compared to face-to-face learning.

> *"My school…said we'll do live lessons, but then a teacher, instead of doing live lessons, just put the PowerPoint online, and then we just had to take notes from that. And they'd say, make four pages of notes on this, do the questions and then that's your work done for the lesson, which wasn't very good, because instead of spending one hour a lesson, I'd spend two or three hours." P68, male, aged 13-17*

The impact of this increased workload on future exams and ultimately grades created an additional source of concern and uncertainty among participants.

> *"I know that the virus has definitely had a big toll on how much I'm thinking about [exams], because there's so much uncertainty… My worries about exams [are] more like: 'how am I going to get the content done in time,' not how am I going to be graded." P49, female, aged 13-17*

Uncertainty about Assessments and Exams

Many participants in the sample discussed feeling stressed, particularly at the start of lockdown, when there was a lack of clear guidance over how schools and universities would manage coursework and exams amid education closures.

> *"University was probably biggest stress at the start because there was an uncertain period when we first went into lockdown, where all the universities were rushing to work out what they were going to do. At this point I was looking at handing in a dissertation and sitting three or four exams, and I didn't know if it was still happening." P54, female, aged 18-22*

When announcements were made that exams would be cancelled, many said that they felt upset or disappointed and reported feeling robbed of the chance to complete their education as planned.

> *"I'm actually really upset that I couldn't sit my exams, so yes. It was a bit of an anticlimax, because I kind of wanted all of the build-up and the apprehension, to finalise, what, two years'*

For peer review only

For peer review only - http://bmjopen.bmj.com/site/about/guidelines.xhtml

worth of… Well, my entire education built up to this. And when it was halted… I found it a bit difficult." P59, female, aged 18-22

Some participants in secondary school reported that they had studied hard outside of school hours to improve their grades even before the pandemic hit and were anxious that this additional effort would not be taken into account when considering their grade predictions.

"Not doing exams, I was gutted. I can't prove myself and I'm being given a letter that they think I could get... they didn't see how hard I was working…they don't see how hard we work outside of school… It's just annoying that we can't prove ourselves." P34, female, aged 13-17

Education Expectations did not match Experience

Many participants spoke of feeling disappointment that their social plans for secondary school or university had not materialised due to closures and disruptions. Symbolic events tied to the start or end of education (such as "Freshers week", "Leavers assembly" or prom) were cancelled and described as another source of loss during the pandemic.

"We didn't get the finale of Year 13 like we normally do where there's like a prom and things like that and it was all just a bit rushed so we didn't get to say bye." P53, male, aged 18-22

Some said that they felt they had "missed out" on an experience that they saw as a rite of passage and one that they had been looking forward to.

"Because we were planning on going out so much and that all being cancelled, it was awful. The day when I was meant to have prom I put my prom dress on just because I wanted to… it was like the idea of it all being cancelled I felt like I was missing out." P55, female, aged 13-17

Lack of Support and Communication

Many participants described feeling that there was a lack of support for their mental health and wellbeing from education providers during the pandemic.

"The school hasn't been great in terms of providing help… the general attitude is: 'it's okay not to be okay, but we're not going to do anything about it.' I know a couple of other schools have a similar situation where you feel like, do they know that you're struggling, but they're not going to do anything." P61, female, aged 13-17

In cases where support was offered, some said that they had experienced this as performative rather than genuine.

For peer review only

"And I feel really let down by the school…Our head of year did a wellbeing check and it didn't even seem genuine, it just seemed like she had to do it for her ego or something like that..." P34, female, aged 13-17

Others described poorly managed communication and a lack of information from education providers about pandemic-related changes, which left students uncertain about their options for rearranging assessments and coursework.

"It would have been nice if I could speak to my personal tutor or something about it, but I think everyone was so busy at the beginning trying to record lectures and put them online, and everything was a bit hectic. So, if there was support it wasn't very clear or easy to find," P64, female, aged 18-22

Poor communication and a perceived lack of support from education providers also resulted in some students feeling forgotten about or unfairly treated. This led to feelings of anger, frustration and a sense that their experiences were not valued.

"At the beginning, kind of March to May period, the response was absolutely shambolic and pretty malevolent to be honest…telling people to go home at their own expense up to and including sort of international students being told that they had to leave within a week even if that meant that their flights were two grand or something." P56, male, aged 18-22

Theme Two: Missing Social Contact During Lockdown

Many participants reported a decline in wellbeing due to feeling lonely and isolated, particularly at the start of lockdown. Being unable to see friends or family and feeling that they were missing out on important life events was a source of sadness and frustration. Some withdrew themselves even further from online social activities after government restrictions were imposed.

Struggling with Isolation

Most participants said that their mental health was affected by social distancing restrictions. Many described this as being due to feeling socially restricted or "trapped" at home and being unable to maintain their usual activities and relationships.

"I'm not a very anxious person. I've struggled with mental health anyway, but… I think in the beginning of lockdown, being trapped at home wasn't ideal in terms of my mental health." P59, female, aged 18-22

For peer review only - http://bmjopen.bmj.com/site/about/guidelines.xhtml

The feeling of being inside and at home for an indeterminate period of time was overwhelming for some, leading to a desire to overcompensate for what was perceived as “lost time” when restrictions began to ease after the first wave of the virus.

> *“As soon as restrictions were relaxed enough that you could meet someone socially distanced, I was down at my friends’ houses that are 100m down the road, three or four times a week, just to get out of my house. I got so frustrated being cooped up.” P55, female, aged 13-17*

The social distancing restrictions were especially challenging for participants who had prior mental health concerns, many of whom reported that their wellbeing had declined while they were socially isolating.

> *“…during lockdown, I got horrendously depressed. So, I’ve had stuff going on mentally, but I was finding it tough. The social isolation made everything crash really badly.” P39, male, aged 18-22*

Social Withdrawal and Loneliness

While many participants had adapted their lives in response to social distancing restrictions, some, particularly adolescents, described socialising less during the lockdown and reported feelings of loneliness or social withdrawal.

> *“I don’t really speak to much of my mates as I used to. It’s made me quite distanced with quite a lot of people.” P66, female, aged 13-17*

At the start of the lockdown, some participants felt uncertain or overwhelmed by the move to online social interactions and made a conscious decision to withdraw from this method of contact.

> *“Before lockdown, I never used video calls or phoning people anyway. I spoke to people through messaging a lot, but I never used Zoom. I never heard of it to be honest. So, when I was suddenly forced to do that, it was just too much, so I’ve stopped talking to people.” P20, male, aged 13-17*

Boredom was commonly reported by a majority of participants and lowered their motivation to interact with others. As the pandemic went on, some participants said that they had nothing to talk about with their peers, or that friends were uninterested in keeping in contact. Therefore, efforts to maintain social connection ceased and usual social ties further disrupted.

> *“I definitely tried to keep in touch with my school friends more at the start, but then it felt very one-sided, so I stopped making all the effort and I haven’t spoken to a few of them in a few months.” P44, female, aged 13-17*

For peer review only - http://bmjopen.bmj.com/site/about/guidelines.xhtml

Participants described feeling lonely throughout different stages of the pandemic, as though the world felt smaller from being at home during the lockdown without any social contact.

> *"There was some socialising over Zoom but overall, I think my world just sort of contracted fairly significantly…" P56, male, aged 18-22*

Several participants felt lonely even in the presence of others as restrictions began to ease (but rules such as mask-wearing and no touching remained in place), with many associating their feelings of loneliness with a lack of physical contact and closeness.

> *"So, even though we are back at school, we're still struggling through the same things. We're still feeling lonely, that we're not able to have that physical contact, which I'm looking forward to having if the pandemic gets better." P51, female, aged 13-17*

Theme Three: Changes to Social Relationships

Relationship Tensions

Participants spoke of stress, anxiety and anger regarding family and friendship tensions during the pandemic. For some, this was due to not being permitted to see their friends, which led to relationship breakdowns.

> *"What's changed is it's put a lot of pressure on relationships, like with my boyfriend and a lot of people I know of have broken up with their boyfriends or girlfriends because of this quarantine and not being able to see them every day, or be around them as much." P19, female, aged 13-17*

For others, differences in opinions between friends led to arguments over what was acceptable or not when following the social distancing rules.

> *"One of my mates, my mate, I actually ended up having an argument, because she was, like, oh, you've got to come and see me." P66, female, aged 13-17*

Participants living with family members said that they experienced difficulties getting along with parents and siblings during lockdown, particularly when living together in small homes or when everyone was working from home.

> *"I don't always get on with my family very well and we would be in each other's faces a hell of a lot and that got very difficult at times… I'd say it gets worse when we have to spend more time together." P38, male, aged 18-22*

For peer review only - http://bmjopen.bmj.com/site/about/guidelines.xhtml

> *"I think everyone just got a little bit stir crazy seeing the same walls and the same people." P44, female, aged 13-17*

Relationship Unity and Connectedness

Despite relationship tensions, participants also described feeling closer with their family members throughout the pandemic and specifically during lockdown periods spent together.

> *"My relationship with my parents is great and it's improved. It was at a great point this year and I think better than it's ever been. And I think lockdown was a good time just to spend more time with them." P37, female, aged 20-24*

Many were reminded about the importance of family and became resolute to make more effort to connect with others in the future.

> *"I won't take for granted as much, like just standing outside talking to my neighbour, phone calls with my [relative]. Just the little things like that. Because obviously it's so easy in normal life just to brush them aside and say, 'oh, I'll ring my [relative] later.' So now I take the time to ring my [relative] and talk to her." P33, female, aged 13-17*

Some participants had "difficult" conversations with family during the lockdown that they otherwise might not have had, for instance, what they would do if a family member became ill with the virus. Having frank and open conversations helped some to feel more connected with their family members than before the pandemic.

> *"I definitely think [the pandemic] forced us to talk about things that we wouldn't always want to talk about. We had to discuss as a family what would happen to my dad if he was to get ill…We discussed those things already in general because we are quite open about that but I think it increased the intensity and frequency of those conversations about death and what would happen and health and things like that… it's helped us to be more open with each other" P41, female, aged 20-24*

Adapting to Maintain Social Connections

In response to social distancing measures, participants described the ways in which they had adapted their social lives during the pandemic, including trying new methods of online communication.

> *"The idea of not knowing when you're next going to be in a crowded place, really able one on one to chat to people I think has just made people search for other ways of connecting with people and that's online." P41, female, aged 20-24*

For peer review only - http://bmjopen.bmj.com/site/about/guidelines.xhtml

Some said they were using dating apps, chatting through gaming platforms and sharing COVID-related "memes" as a way "to cope with difficult things" and enhance their sense of connectedness with others. Older participants in particular said that they were more focused on online dating during lockdown as a way to connect: *"I joined a dating app just to meet people."* And others sought out dating apps as a way of alleviating boredom.

> *"I personally don't think I would've downloaded that [dating] app if it hadn't have been for the pandemic because I've tried them before and really didn't like them and hadn't had much success. And then I was just bored and felt like when am I next going to meet someone?... I feel like that probably wouldn't have happened if it wasn't for lockdown." P77, female, aged 20-24*

New friendships were formed during lockdown with the help of chat functions on gaming and social media platforms (i.e., "Whatsapp").

> *"I have made a bunch of friends that are from online around my age from playing a game...so during the pandemic instead of chatting with my friends I'd be playing a game with them almost every day. Well, every day for a few hours chatting, having fun which was nice." P21, male, aged 13-17*

Changes in Feelings about Socialising after Lockdown

Around half of participants described how their feelings about in-person social interactions had changed since the start of the pandemic. Some described this in terms of feeling more vigilant about being around other people and of the risks associated with future virus transmission.

> *"I think I'm going to be a lot more cautious about other people, obviously, like people you don't know being quite close to you, physically." P45, male, aged 13-17*

Although individual concern about their own risk of catching COVID was not widespread in the group overall, some participants still reported feeling safer during lockdown away from other people and less exposed to the perceived risk of catching COVID-19.

> *"I felt less anxious about everything when we were in full lockdown and that the lifting out of lockdown, that's quite scary in many ways for many different people…obviously introducing more risk into your life, whereas when everyone was in full lockdown, I feel like there was something quite comforting in a weird way about that." P37, female, aged 20-24*

Adolescents in particular said they felt apprehensive about future social situations from not having been exposed to "*real life*" and becoming accustomed to life in lockdown.

For peer review only - http://bmjopen.bmj.com/site/about/guidelines.xhtml

"I just feel like I've lost the ability to form coherent sentences. I know that sounds really stupid. It's just been really hard." P34, female, aged 13-17

Concern about having to socialise was especially present in those returning to secondary school after months in lockdown, following difficulties in keeping in touch with friends and maintaining positive friendships.

"I was nervous, because a lot changed during quarantine. I think people got really tetchy staying at home, so there were a lot of arguments. Loads of people fell out of contact with one another…So, it was harder for us to find common things to talk about, we were just speaking about the same topics again and again. So, going back [to school, I] was really nervous because we didn't know what we would talk about." P49, female, aged 13-17

Theme Four: Improved Wellbeing During Lockdown

For those who experienced busy and stressful lives prior to the pandemic, social distancing restrictions provided a chance to pause and reflect, with resultant mental health benefits. Having more choice about how to spend free time was described by participants as relaxing and removed previous sources of stress. For some, their wellbeing was enhanced by support from others and an increased public awareness of mental health self-management during lockdown.

Improved Mental Health Compared to Pre-pandemic

In comparison to a majority of participants who reported struggling with their wellbeing during the pandemic, a smaller group of adolescents described how their mental health had improved during the first lockdown.

"Well, before lockdown, my mental health wasn't great… my sleep schedule was all over the place, school stress, but actually, lockdown gave me a chance to focus and make myself a lot better in terms of the positive emotions" P49, female, aged 13-17

They described busy or stressful school lives prior to the pandemic. School closures and a shift to online learning, particularly for those who were not preparing for their exams, meant that some had less work to do. Social distancing restrictions and pandemic-related closures therefore provided an opportunity to relax and reflect.

"I think it's been just I guess inconvenience rather than a full crisis in my eyes. It probably should have affected me more but, yes, I quite like it in quarantine to be honest. I don't have to get up, I can just chill around. It's a lot nicer than having to rush around constantly." P21, male, aged 13-17

For peer review only - http://bmjopen.bmj.com/site/about/guidelines.xhtml

As previously reported, the closure of schools and universities meant an increased workload for some, but the flexibility of choosing what to do between lessons and during free time meant the lockdown felt "*like a really long summer holiday*" for others.

> *"I was happy when school got cancelled… it was still hard to do the work at home, but it was just because it was just like, in between I could just do whatever I wanted, so it was just better, sometimes I just go and play in the garden, football, or just be on my phone or the PS4." P28, male, aged 13-17*

Increased Self-Awareness

Throughout the course of the pandemic, some participants had more time to take up hobbies and exercise, but several young adults also commented on the personal growth and increased self-awareness that they had observed.

> *"[This experience has] taught me to take everything a day as it comes. And then see what happens. But I won't be surprised if, in a good way, that things change and it happens for the better.… it allowed me to challenge myself more... now things have started to flow better with how I am as a person." P60, female, aged 18-22*

Some participants reflected on the stark changes in their lives during the pandemic compared with pre-pandemic and said they felt differently about how they wanted their life to be when social distancing restrictions eased.

> *"First, I think someone hit the brakes, I think, like everything stopped…[Life is] no longer that constant, hectic mess of going out…overall, it's a lot less intense maybe, now." P39, male, 18-22*

Increased Awareness of Mental Health

Some participants said they felt an increased awareness of their own mental health and how to protect it during the pandemic, through initiatives driven by workplaces and education providers.

> *"My organisation did allocate three weeks of wellbeing leave which is a really interesting move. And that was on top of annual leave. And that was complementary leave because the reason why they did it is that they said that it's a really hard time and they just think everyone needs the break." P37, female, aged 20-24*

Some said they had not paid attention to their mental health in the past compared to now, having been through a pandemic and enforced social isolation.

For peer review only - http://bmjopen.bmj.com/site/about/guidelines.xhtml

"[the pandemic has] shown that health and mental health and mental state is important. As it always was, but I don't think I realised it as much as I would now." P46, male, aged 13-17

This perceived change in mental health awareness helped make it feel easier to talk to others about their wellbeing, and for some, this "opened up" a dialogue with friends and family that had never been achieved before.

"It opened up really big conversations within our family about mental health that I don't think they've ever had before like the generations. So those are two really big positives for me I think. Both myself and opening that up wider...It was just conversations that we'd never had before." P37, female, aged 20-24

Accessing Mental Health Support

Some participants sought support to help cope with pandemic-related distress, either informally through friends and family, or formally through education providers, health services, or employers.

"I did talk to one of my teachers actually at school, on Teams…And she helped me and a lot of friends about things to do during lockdown." P50, female, aged 13-17

In some cases, regular offers of support and check-ins by educational staff were sufficiently reassuring to know that help was on hand if needed.

"[University staff would] email every week, checking in. They gave me two… deadline extensions, for my work, which helped me a lot. And my tutor was really helpful. He did check in every couple of weeks to make sure I was all right. Even over the summer, when they haven't necessarily been at uni. So, that was good." P57, female, aged 18-22

Discussion

We sought the views of adolescents and young adults about their wellbeing and social lives during the COVID-19 pandemic using detailed qualitative interviews. Young people described mental health consequences associated with education closures and lockdown measures, including loneliness, frustration, a sense of loss about cancelled plans and assessments, and uncertainty about socialising in the future. Our findings highlight how some felt paradoxically isolated but also hesitant about reintegrating with others as the lockdown restrictions ease. For some, the pandemic and associated restrictions provided a platform for new relationships to develop and existing relationships to strengthen, while for others, social isolation added strain and divisions. Despite the challenges, we found evidence of a potentially positive change during the pandemic, including an increased

For peer review only

For peer review only - http://bmjopen.bmj.com/site/about/guidelines.xhtml

awareness of mental health self-management, and having more open conversations about mental health compared to pre-pandemic.

While the long-term effect of school closures on young people's health, income and productivity is yet to be fully realised, [29] disruptions to education on this scale have not occurred since the second World War. [30] These changes will likely have long term consequences requiring ongoing support, [31] with particular attention needed on how sociodemographic factors have affected young peoples' experiences during the pandemic. [32] Around 60% of students living in affluent areas had access to online learning during the pandemic, compared with 23% of students living in more deprived areas. [33] A loss of learning has already been observed by teaching staff in the UK, [34] and our findings suggest this may be linked to increased workloads, sharing small workspaces at home, and difficulties with concentration. If unaddressed, this may restrict opportunities for employment and social mobility available to young people in the future. [31] It is essential that those without the resources to work optimally from home are supported, to ensure that educational and social inequalities are not exacerbated, both as COVID-19 persists and in other future scenarios where remote learning may be required.

It is estimated more than half of students have experienced adverse psychological wellbeing during the COVID-19 pandemic. [35] We found that paradoxically, while some participants felt isolated and lonely, they were also apprehensive about social distancing restrictions easing and life returning to "normal". Graded or phased approaches for those returning to education [36] and employment are recommended as lockdown restrictions ease. [37] Academic challenges have been frequently reported by young people during the pandemic, particularly among secondary school aged youth. [32] We also noted concern among those who had access to online learning but were unable to sit exams and finish their coursework. Several young people felt they did not demonstrate their full potential when their exams were cancelled and did not want to be known as the generation who were given a "free pass". Scott et al. also found young people reported feeling less confident and like an "imposter" following cancelled exams due to the pandemic. [38] Teaching staff may be well-placed to address these concerns by clearly communicating revised assessment plans and providing reassurance about the assessment process for future students affected by pandemic-related education disruptions. Teachers also require clear guidance and coordination from government and educational authorities to be able to deliver these messages, [34] which were absent during the first wave of educational establishment closures.

Young people have reported some of the highest rates of loneliness during the pandemic, [39] which have been associated with a decline in psychological wellbeing. [40] Initiatives that enable young

For peer review only - http://bmjopen.bmj.com/site/about/guidelines.xhtml

people to easily connect with others and access support for their mental health while adhering to social distancing guidelines are greatly needed. [40] Participants in our study frequently cited mental health consequences due to social isolation and loneliness, which some used gaming platforms, social media, and online dating apps to help address. These findings build on those shown in previous research involving young people during the first national lockdown, [41] including the sense of loss and uncertainty frequently reported by this group. Our results build on this by suggesting how social isolation led to these effects, including what felt like missed opportunities to create memories with friends and missed social events seen as rites of passage in young adulthood.

The effects of isolation and loneliness for young people are likely to persist for some time, even after social distancing restrictions have lifted. [42] The path ahead will require additional resources for education providers and coordinated efforts from policy makers, education authorities, and health and social care to provide reassurance that service provision is responsive to the needs of young people. [43] This response should be formulated in collaboration with young people to ensure that any future support incorporates appropriate coping strategies and protective activities, [44] such as those identified by our study, including check-ins from education providers and wellbeing initiatives from employers. These responses could help to validate the unique struggles that many young people have faced throughout the different stages of the pandemic. [44]

Despite the many negative effects of the pandemic on mental health in young people identified to date, evidence also suggests that young people's altruistic motivations [45,46] and prosocial behaviours have increased during the pandemic, including providing practical support to others and donating to charities perceived to be in need. [47] It is encouraging that some participants in this study reported feeling more able to talk about their mental health compared with pre-pandemic, suggesting less social stigma perceived by young people in this study to be associated with mental health and wellbeing. This was, at times, facilitated by top-down efforts from education providers, employers, and parents. In this study and others, [20] we found that many have been reminded of the importance of family and friends during adversity. These changes represent a potentially positive side effect of the pandemic, providing they are supported and maintained.

Strengths and Limitations

To our knowledge, this is the first UK study to use qualitative interviewing methods to learn more about the mental health and wellbeing of young people (aged 13-24) during multiple phases of the COVID-19 pandemic. Purposive recruitment strategies were used to try and increase the variability of experiences within the group of participants interviewed, but despite this, our sample contained more 15- and 16-year-olds than other age groups, and participants who identified as White than other

For peer review only - http://bmjopen.bmj.com/site/about/guidelines.xhtml

ethnic groups. Those whom we spoke with were from a range of living situations and health statuses; however, there were some data we did not collect that is likely to have influenced participant experiences during the pandemic, including socioeconomic status, sexual orientation, and gender expression. Data were gathered throughout several key time points of the UK pandemic response, including as schools were reopening after the first lockdown, and as the second wave of the virus had begun. We explored a range of issues during interviews that were pertinent to the research questions, but some topics identified during this study could be investigated in further depth by future researchers, including the impact of the COVID-19 pandemic on intimate relationships among adolescents and young adults. We relied on participants self-identifying to take part in the research, which may have meant that the sample whose experiences were more positive than those less comfortable discussing their experiences or with less time to take part in research. Remote interviewing techniques may have excluded some groups with limited online access; however, we also offered to conduct telephone calls and remote data collection methods enabled us to speak to people from across the UK.

Conclusions

When the pandemic began and restrictions were introduced, many young people lost a sense of certainty, community and belonging provided by their routines, social circles, and education providers. Our findings highlight how support for adolescents and young people to develop and maintain their social connections and access mental health support is needed even after the virus is controlled. Findings suggest that young people in the study may have felt less stigma talking about their mental health now compared to pre-pandemic, which was in part facilitated by initiatives through schools, universities, and employers. Most participants in our study struggled with their wellbeing and loneliness during the pandemic, and our results highlight the vital role of education providers giving regular guidance and support to help alleviate their concerns. Many young people we spoke with are worried about how the pandemic has affected their education and social connections, and support for young people should be tailored accordingly around these concerns. We also highlight the detrimental impact that an absence of information and support had on young people's mental health. More must be done to ensure offers of support to young are genuine and reinforced with signposting to information, advice and services when young people do reach out for help.

References

1. World Health Organisation. Coronavirus disease 2019 (COVID-19) Situation Report – 51 [Internet]. 2020. Available from: https://www.who.int/docs/default-source/coronaviruse/situation-reports/20200311-sitrep-51-covid-19.pdf?sfvrsn=1ba62e57_10

For peer review only - http://bmjopen.bmj.com/site/about/guidelines.xhtml

2. Niedzwiedz CL, Green MJ, Benzeval M, Campbell D, Craig P, Demou E, et al. Mental health and health behaviours before and during the initial phase of the COVID-19 lockdown: longitudinal analyses of the UK Household Longitudinal Study. J Epidemiol Community Health. 2020 Sep 25;jech-2020-215060.

3. Pierce M, Hope H, Ford T, Hatch S, Hotopf M, John A, et al. Mental health before and during the COVID-19 pandemic: a longitudinal probability sample survey of the UK population. Lancet Psychiatry. 2020 Oct;7(10):883–92.

4. Sprang G, Silman M. Posttraumatic Stress Disorder in Parents and Youth After Health-Related Disasters. Disaster Med Public Health Prep. 2013 Feb;7(1):105–10.

5. Brooks SK, Webster RK, Smith LE, Woodland L, Wessely S, Greenberg N, et al. The psychological impact of quarantine and how to reduce it: rapid review of the evidence. The Lancet. 2020 Mar;395(10227):912–20.

6. Kwong ASF, Pearson RM, Smith D, Northstone K, Lawlor DA, Timpson NJ. Longitudinal evidence for persistent anxiety in young adults through COVID-19 restrictions. Wellcome Open Res. 2020 Aug 19;5:195.

7. O'Sullivan K, Clark S, McGrane A, Rock N, Burke L, Boyle N, et al. A Qualitative Study of Child and Adolescent Mental Health during the COVID-19 Pandemic in Ireland. Int J Environ Res Public Health. 2021 Jan 25;18(3):1062.

8. Bhopal SS, Bagaria J, Olabi B, Bhopal R. Children and young people remain at low risk of COVID-19 mortality. Lancet Child Adolesc Health. 2021 Mar 11;

9. Ludvigsson JF. Systematic review of COVID-19 in children shows milder cases and a better prognosis than adults. Acta Paediatr. 2020 Jun;109(6):1088–95.

10. Fegert JM, Vitiello B, Plener PL, Clemens V. Challenges and burden of the Coronavirus 2019 (COVID-19) pandemic for child and adolescent mental health: a narrative review to highlight clinical and research needs in the acute phase and the long return to normality. Child Adolesc Psychiatry Ment Health. 2020 Dec;14(1):20.

11. Fancourt D, Steptoe A, Bu F. Trajectories of anxiety and depressive symptoms during enforced isolation due to COVID-19 in England: a longitudinal observational study. Lancet Psychiatry [Internet]. 2020 Dec [cited 2020 Dec 11]; Available from: https://linkinghub.elsevier.com/retrieve/pii/S221503662030482X

12. Dewa LH, Crandell C, Choong E, Jaques J, Bottle A, Kilkenny C, et al. CCopeY: A Mixed-Methods Coproduced Study on the Mental Health Status and Coping Strategies of Young People During COVID-19 UK Lockdown. J Adolesc Health. 2021 Apr;68(4):666–75.

13. Lee J. Mental health effects of school closures during COVID-19. Lancet Child Adolesc Health. 2020 Jun;4(6):421.

14. Ganson KT, Tsai AC, Weiser SD, Benabou SE, Nagata JM. Job Insecurity and Symptoms of Anxiety and Depression Among U.S. Young Adults During COVID-19. J Adolesc Health. 2021 Jan;68(1):53–6.

15. Romanou E, Belton E. Isolated and struggling: Social isolation and the risk of child maltreatment, in lockdown and beyond. [Internet]. 2020. Available from:

For peer review only - http://bmjopen.bmj.com/site/about/guidelines.xhtml

https://learning.nspcc.org.uk/media/2246/isolated-and-struggling-social-isolation-risk-child-maltreatment-lockdown-and-beyond.pdf

16. Young Minds. Coronavirus: Impact on young people with mental health needs. [Internet]. 2021. Available from: https://youngminds.org.uk/media/4350/coronavirus-report-winter.pdf

17. Waite P, Pearcey S, Shum A, Raw J, Patalay P, Creswell C. How did the mental health of children and adolescents change during early lockdown during the COVID-19 pandemic in the UK? [Internet]. PsyArXiv; 2020 Dec [cited 2021 Feb 26]. Available from: https://osf.io/t8rfx

18. Bu F, Steptoe A, Mak HW, Fancourt D. Time-use and mental health during the COVID-19 pandemic: a panel analysis of 55,204 adults followed across 11 weeks of lockdown in the UK. 2020; Available from: https://www.medrxiv.org/content/10.1101/2020.08.18.20177345v1

19. Campbell S, Greenwood M, Prior S, Shearer T, Walkem K, Young S, et al. Purposive sampling: complex or simple? Research case examples. J Res Nurs. 2020 Dec;25(8):652–61.

20. Aughterson H, McKinlay AR, Fancourt D, Burton A. Psychosocial impact on frontline health and social care professionals in the UK during the COVID-19 pandemic: a qualitative interview study. BMJ Open. 2021 Feb 1;11(2):e047353.

21. Burton A, McKinlay AR, Aughterson H, Fancourt D. Impact of the Covid-19 pandemic on the mental health and wellbeing of adults with mental health conditions in the UK: A qualitative interview study. Medrxiv Prepr Serv Health Sci [Internet]. 2020; Available from: https://www.medrxiv.org/content/10.1101/2020.12.01.20241067v1

22. May T, Aughterson H, Fancourt D, Burton A. 'Stressed, uncomfortable, vulnerable, neglected': a qualitative study of the psychological and social impact of the COVID-19 pandemic on UK frontline keyworkers. SocArXiv [Internet]. 2021; Available from: https://osf.io/preprints/socarxiv/dn43c/

23. Antonovsky A. Health, stress, and coping. San Francisco, US: Jossey-Bass; 1979.

24. Berkman LF, Glass T, Brissette I, Seeman TE. From social integration to health: Durkheim in the new millennium. Soc Sci Med. 2000 Sep;51(6):843–57.

25. Arnett JJ. Emerging adulthood: A theory of development from the late teens through the twenties. Am Psychol. 2000;55(5):469–80.

26. Elliott V. Thinking about the Coding Process in Qualitative Data Analysis. Qual Rep [Internet]. 2018 Nov 24 [cited 2021 Sep 30]; Available from: https://nsuworks.nova.edu/tqr/vol23/iss11/14/

27. Braun V, Clarke V. Reflecting on reflexive thematic analysis. Qual Res Sport Exerc Health. 2019 Aug 8;11(4):589–97.

28. Braun V, Clarke V. One size fits all? What counts as quality practice in (reflexive) thematic analysis? Qual Res Psychol. 2020 Aug 12;1–25.

29. Viner RM, Russell SJ, Croker H, Packer J, Ward J, Stansfield C, et al. School closure and management practices during coronavirus outbreaks including COVID-19: a rapid systematic review. Lancet Child Adolesc Health. 2020 May;4(5):397–404.

For peer review only - http://bmjopen.bmj.com/site/about/guidelines.xhtml

30. d'Orville H. COVID-19 causes unprecedented educational disruption: Is there a road towards a new normal? PROSPECTS. 2020 Oct;49(1–2):11–5.

31. The British Academy. THE COVID DECADE Understanding the long-term societal impacts of COVID-19 [Internet]. 2021. Available from: https://www.thebritishacademy.ac.uk/documents/3238/COVID-decade-understanding-long-term-societal-impacts-COVID-19.pdf

32. Scott SR, Rivera KM, Rushing E, Manczak EM, Rozek CS, Doom JR. "I Hate This": A Qualitative Analysis of Adolescents' Self-Reported Challenges During the COVID-19 Pandemic. J Adolesc Health. 2021 Feb;68(2):262–9.

33. Cullinane C, Montacute R. COVID-19 and Social Mobility Impact Brief #1: School Closures [Internet]. 2020. Available from: https://www.suttontrust.com/wp-content/uploads/2021/01/School-Shutdown-Covid-19.pdf

34. Kim L, Leary R, Asbury K. 'We need clear directions, if we're going to move forward. It's as simple as that': Teachers' narratives during partial school reopenings in the COVID-19 pandemic. PsyArXiv. 2021;

35. Office for National Statistics. Coronavirus and the impact on students in higher education in England: September to December 2020 [Internet]. 2020. Available from: https://www.ons.gov.uk/peoplepopulationandcommunity/educationandchildcare/articles/coronavirusandtheimpactonstudentsinhighereducationinenglandseptembertodecember2020/2020-12-21

36. Viner RM, Bonell C, Drake L, Jourdan D, Davies N, Baltag V, et al. Reopening schools during the COVID-19 pandemic: governments must balance the uncertainty and risks of reopening schools against the clear harms associated with prolonged closure. Arch Dis Child. 2021 Feb;106(2):111–3.

37. Habersaat KB, Betsch C, Danchin M, Sunstein CR, Böhm R, Falk A, et al. Ten considerations for effectively managing the COVID-19 transition. Nat Hum Behav. 2020 Jul;4(7):677–87.

38. Scott S, McGowan VJ, Visram S. 'I'm Gonna Tell You about How Mrs Rona Has Affected Me'. Exploring Young People's Experiences of the COVID-19 Pandemic in North East England: A Qualitative Diary-Based Study. Int J Environ Res Public Health. 2021 Apr 6;18(7):3837.

39. Bu F, Steptoe A, Fancourt D. Loneliness during a strict lockdown: Trajectories and predictors during the COVID-19 pandemic in 38,217 United Kingdom adults. Soc Sci Med. 2020 Nov;113521.

40. Lee C, Cadigan C, Rhew I. Increases in Loneliness Among Young Adults During the COVID-19 Pandemic and Association With Increases in Mental Health Problems. J Adolesc Health. 2020;

41. Demkowicz O, Ashworth E, O'Neill A, Hanley T, Pert K. Teenagers' Experiences of Life in Lockdown: The TELL study briefing. 2020; Available from: https://www.seed.manchester.ac.uk/education/research/impact/teenagers-experiences-of-life-in-lockdown/

42. Loades ME, Chatburn E, Higson-Sweeney N, Reynolds S, Shafran R, Brigden A, et al. Rapid Systematic Review: The Impact of Social Isolation and Loneliness on the Mental Health of

For peer review only

Children and Adolescents in the Context of COVID-19. J Am Acad Child Adolesc Psychiatry. 2020 Nov;59(11):1218-1239.e3.

43. Kim LE, Asbury K. 'Like a rug had been pulled from under you': The impact of COVID-19 on teachers in England during the first six weeks of the UK lockdown. Br J Educ Psychol. 2020 Dec;90(4):1062–83.

44. Efuribe C, Barre-Hemingway M, Vaghefi E, Suleiman AB. Coping With the COVID-19 Crisis: A Call for Youth Engagement and the Inclusion of Young People in Matters That Affect Their Lives. J Adolesc Health. 2020 Jul;67(1):16–7.

45. August R, Dapkewicz A. Benefit finding in the COVID-19 pandemic: College students' positive coping strategies. J Posit Sch Psychol. 2020 Dec 8;5(2):73–86.

46. Fisher H, Lambert H, Hickman M, Yardley L, Audrey S. Experiences of the coronavirus disease-19 (COVID-19) pandemic from the perspectives of young people: Rapid qualitative study. Public Health Pract. 2021 Nov;2:100162.

47. Alvis L, Douglas R, Shook N, Oosterhoff B. Associations between Adolescents' Prosocial Experiences and Mental Health During the COVID-19 Pandemic. PsyArXiv [Internet]. 2020; Available from: https://psyarxiv.com/2s73n

For peer review only

For peer review only - http://bmjopen.bmj.com/site/about/guidelines.xhtml

Figure 1 Interview Guide Examples

- *How do you feel about the changes that have been brought about by Covid-19?*
- *Have they had any impact on your mental health or wellbeing?*
- *What are the things most bothering you at the moment?*
- *Has the pandemic meant that you have any worries for the future?*

209x297mm (300 x 300 DPI)

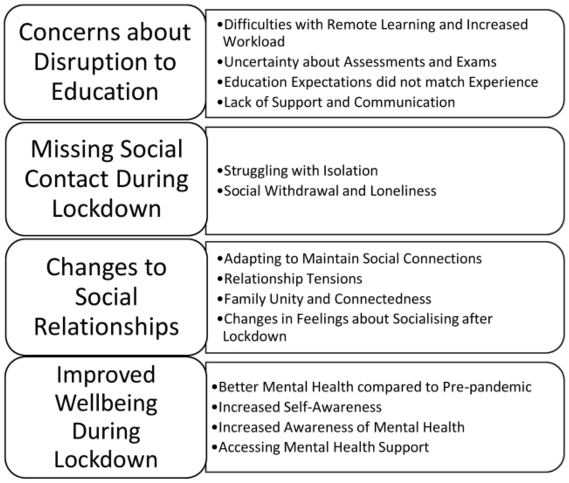

Figure 2 Themes and sub-themes

209x297mm (300 x 300 DPI)

Supplementary Material 1: Topic Guide

Please could you describe what normal life was like for you before the pandemic and the social distancing? (can explain before – e.g. staying at home, stopping visiting people's houses, having to stand 2 metres apart, etc)

- Who do you normally live with separated/ extended family?
- Do you have any siblings/ pets?
- School type, do you have friends you would normally visit or would visit you – please tell me about this?
- Do you have any hobbies or things you enjoyed doing out of school time?

UNDERSTANDING AND ADHERENCE TO GUIDELINES

At the moment, are you 'staying at home'?

- Can you tell me what this is like for you and your family?
  - i.e. are you/ your parents going out for shopping or for walk/ exercise
  - Or staying inside with no outside exercise?/ other people dropping off shopping?

- **What do you understand by the 'social distancing' advice that is being given – what does it mean to you? (Ask to list the rules they are aware of)**
- Have you been…
  - Avoiding crowds
  - Keeping distance from others
  - Isolating (staying inside)
  - Avoiding close contact greetings
  - Socialising/going out only with those in your household

Have you been able to stick to the social distancing advice that has been given? Please tell us about why/ why not?

- What helps you to stick to it?
- Or – what are you finding difficult about sticking to the rules?
- What has motivated you to do this?
- Is there anything that makes it easier to do or harder

[COM-B prompts can be used here, to include:]

- Any existing physical or mental health problems
- Group membership/ applicability Whether it feels like the rules apply to you
- Beliefs about whether it will keep you healthy
- Beliefs about whether it will keep other people healthy
- Having to go out for things like food
- Work/ Caring responsibilities, providing emotional support
- Friends suggesting meeting, family or parents wanting to go out
- Government rules/punishments,
- Feelings about losing normal life
- Change of routine/ habits

For peer review only - http://bmjopen.bmj.com/site/about/guidelines.xhtml

SOCIAL LIFE – In terms of before the pandemic, How would you describe your social life before the Covid-19 pandemic started?
Although some of the question seems less directly relevant, we are trying to understand a big picture of how groups affect/ influence how we feel.

- How would you describe the group of people you knew before the pandemic (these may be the same as now)
  - Are they friends or family or both mainly?
  - Do they live near you or far away?
  - How often did you see each other?
- Did you speak to family and friends face to face/online?

- Were you involved in any groups outside school – please describe (examples such as choir, dancing classes, football teams, youth groups/ youth theatre, Scouts/Guides, social activities etc)
- Did you have people who helped you, such as teachers, Scout leaders, parents, grandparents, family friends? How did they help you, for example listening, help with homework, help with mental health?
- Can you tell us about whether or not your friendship groups encouraged you to get involved in things? Did you find you compared your life to theirs?
- Social roles possibly as a young carer/ older sibling

How would you describe your social life now because of Covid-19?

Please tell us about this – make clear that we are talking about after the start of the pandemic, to see if anything has changed.

Prompts include:

- How would you describe the group of people you know now?
  - Are they friends or family or both mainly?
  - Do they live near you or far away?
  - How often did you see each other?
- Do you speak to family and friends face to face/online?
- Are you seeing or speaking to teachers and students as part of school online?

- Are you involved in any online groups outside school – please describe (examples such as online choir, dancing classes, , youth groups/ youth theatre, Scouts/Guides, social activities etc)
- Do you have people who help you, such as teachers, Scout leaders, parents, grandparents, family friends? How did they help you, for example listening, help with homework, help with mental health?
- Can you tell us about any ways your friendship groups influence you such as encouraging you to get involved in things? Do you find you compare your life and theirs?
- Social roles possibly as a young carer

For peer review only - http://bmjopen.bmj.com/site/about/guidelines.xhtml

MENTAL HEALTH

How do you feel about the changes that have been brought about by Covid-19?
Have they had any impact on your mental health or wellbeing? Please tell us about these

- What are the things most bothering you at the moment?
- Have you experienced any impact on positive emotions? (prompts: concentration, being able to do and achieve things, relationships with others, how well you are managing and feelings of control over your situation?)
- Have you experienced any negative feelings? (prompts: such as, not enjoying things as much, anxiety, worry)
- Could you tell us if you have had any physical symptoms that might be due to being stressed or anxious? (prompts: fatigue, sleep problems, pain, illness symptoms, heart racing)

Have you been doing/ planning anything to help with this?

- Connecting with family or friends online
- Online groups?
- Hobbies/ Reading
- Exercise at home/ outside the home
- Volunteering
- Anything else you can tell me about?

Why are you doing/ not doing these things?

- Helpful/ not helpful – please tell us why
- Enjoyable
- Good for mental health/ wellbeing
- Can't get online, not connected, not comfortable, affordability, confidence in using/ skills
- Skills in using the internet/ communication software
- Living arrangements/ Work/ caring demands
- Peer support/ pressure
- Difficulties/ restriction in physical environment

PROSPECTION

Has the pandemic meant that you have any worries for the future?

How are these different from the worries you had before?

- Sense of control/ powerlessness
- Severity of worries / perspective

Will this change the way you live your life in future?

- The way you connect with others
- How you look after yourself
- How you support others
-

Has this changed what things are important to you?

For peer review only - http://bmjopen.bmj.com/site/about/guidelines.xhtml

Standards for Reporting Qualitative Research (SRQR)*

http://www.equator-network.org/reporting-guidelines/srqr/

Title and abstract

	Page/line no(s).
**Title** - Concise description of the nature and topic of the study Identifying the study as qualitative or indicating the approach (e.g., ethnography, grounded theory) or data collection methods (e.g., interview, focus group) is recommended	1, 1-3
**Abstract** - Summary of key elements of the study using the abstract format of the intended publication; typically includes background, purpose, methods, results, and conclusions	2, 1-17

Introduction

**Problem formulation** - Description and significance of the problem/phenomenon studied; review of relevant theory and empirical work; problem statement	3, 16-30
**Purpose or research question** - Purpose of the study and specific objectives or questions	4, 1-3

Methods

**Qualitative approach and research paradigm** - Qualitative approach (e.g., ethnography, grounded theory, case study, phenomenology, narrative research) and guiding theory if appropriate; identifying the research paradigm (e.g., postpositivist, constructivist/ interpretivist) is also recommended; rationale**	4, 5-6<br>5, 18-19
**Researcher characteristics and reflexivity** - Researchers' characteristics that may influence the research, including personal attributes, qualifications/experience, relationship with participants, assumptions, and/or presuppositions; potential or actual interaction between researchers' characteristics and the research questions, approach, methods, results, and/or transferability	4, 28-32
**Context** - Setting/site and salient contextual factors; rationale**	4, 28
**Sampling strategy** - How and why research participants, documents, or events were selected; criteria for deciding when no further sampling was necessary (e.g., sampling saturation); rationale**	4, 18-24
**Ethical issues pertaining to human subjects** - Documentation of approval by an appropriate ethics review board and participant consent, or explanation for lack thereof; other confidentiality and data security issues	4, 9-10<br>4, 14-16
**Data collection methods** - Types of data collected; details of data collection procedures including (as appropriate) start and stop dates of data collection and analysis, iterative process, triangulation of sources/methods, and modification of procedures in response to evolving study findings; rationale**	4, 29 (types of data)<br>5, 3-5 (responding to evolving findings)

For peer review only - http://bmjopen.bmj.com/site/about/guidelines.xhtml

	5, 27 – 6, 2 (iterative theme generation)<br>6, 4 (dates)
**Data collection instruments and technologies** - Description of instruments (e.g., interview guides, questionnaires) and devices (e.g., audio recorders) used for data collection; if/how the instrument(s) changed over the course of the study	Topic Guide in Supplementary Data
**Units of study** - Number and relevant characteristics of participants, documents, or events included in the study; level of participation (could be reported in results)	Table on p6, 11-13
**Data processing** - Methods for processing data prior to and during analysis, including transcription, data entry, data management and security, verification of data integrity, data coding, and anonymization/de-identification of excerpts	5, 17-27
**Data analysis** - Process by which inferences, themes, etc., were identified and developed, including the researchers involved in data analysis; usually references a specific paradigm or approach; rationale**	5, 17-27
**Techniques to enhance trustworthiness** - Techniques to enhance trustworthiness and credibility of data analysis (e.g., member checking, audit trail, triangulation); rationale**	5, 20

Results/findings

**Synthesis and interpretation** - Main findings (e.g., interpretations, inferences, and themes); might include development of a theory or model, or integration with prior research or theory	p6-16
**Links to empirical data** - Evidence (e.g., quotes, field notes, text excerpts, photographs) to substantiate analytic findings	p6-16

Discussion

**Integration with prior work, implications, transferability, and contribution(s) to the field** - Short summary of main findings; explanation of how findings and conclusions connect to, support, elaborate on, or challenge conclusions of earlier scholarship; discussion of scope of application/generalizability; identification of unique contribution(s) to scholarship in a discipline or field	17-19
**Limitations** - Trustworthiness and limitations of findings	19, 12-23

Other

**Conflicts of interest** - Potential sources of influence or perceived influence on study conduct and conclusions; how these were managed	1, 16-17
**Funding** - Sources of funding and other support; role of funders in data collection, interpretation, and reporting	1, 18-23

For peer review only

*The authors created the SRQR by searching the literature to identify guidelines, reporting standards, and critical appraisal criteria for qualitative research; reviewing the reference lists of retrieved sources; and contacting experts to gain feedback. The SRQR aims to improve the transparency of all aspects of qualitative research by providing clear standards for reporting qualitative research.

**The rationale should briefly discuss the justification for choosing that theory, approach, method, or technique rather than other options available, the assumptions and limitations implicit in those choices, and how those choices influence study conclusions and transferability. As appropriate, the rationale for several items might be discussed together.

Reference:

O'Brien BC, Harris IB, Beckman TJ, Reed DA, Cook DA. **Standards for reporting qualitative research: a synthesis of recommendations.** *Academic Medicine*, Vol. 89, No. 9 / Sept 2014 DOI: 10.1097/ACM.0000000000000388

For peer review only

For peer review only - http://bmjopen.bmj.com/site/about/guidelines.xhtml

BMJ Open

‘You’re just there, alone in your room with your thoughts:’ A qualitative study about the psychosocial impact of lockdown among young people during the COVID-19 pandemic

Journal:	*BMJ Open*
Manuscript ID	bmjopen-2021-053676.R2
Article Type:	Original research
Date Submitted by the Author:	17-Jan-2022
Complete List of Authors:	McKinlay, Alison; University College London Research Department of Epidemiology and Public Health, Department of Behavioural Science and Health<br>May, Tom; University College London, Behavioural Science and Health, Institute of Epidemiology and Health Care<br>Dawes, Jo; University College London Research Department of Epidemiology and Public Health, Department of Behavioural Science and Health<br>Fancourt, Daisy; University College London Research Department of Epidemiology and Public Health, Department of Behavioural Science and Health<br>Burton, Alexandra; University College London Research Department of Epidemiology and Public Health, Department of Behavioural Science and Health
**Primary Subject Heading**:	Public health
Secondary Subject Heading:	Mental health
Keywords:	QUALITATIVE RESEARCH, COVID-19, MENTAL HEALTH, SOCIAL MEDICINE

SCHOLARONE™
Manuscripts

I, the Submitting Author has the right to grant and does grant on behalf of all authors of the Work (as defined in the below author licence), an exclusive licence and/or a non-exclusive licence for contributions from authors who are: i) UK Crown employees; ii) where BMJ has agreed a CC-BY licence shall apply, and/or iii) in accordance with the terms applicable for US Federal Government officers or employees acting as part of their official duties; on a worldwide, perpetual, irrevocable, royalty-free basis to BMJ Publishing Group Ltd ("BMJ") its licensees and where the relevant Journal is co-owned by BMJ to the co-owners of the Journal, to publish the Work in this journal and any other BMJ products and to exploit all rights, as set out in our licence.

The Submitting Author accepts and understands that any supply made under these terms is made by BMJ to the Submitting Author unless you are acting as an employee on behalf of your employer or a postgraduate student of an affiliated institution which is paying any applicable article publishing charge ("APC") for Open Access articles. Where the Submitting Author wishes to make the Work available on an Open Access basis (and intends to pay the relevant APC), the terms of reuse of such Open Access shall be governed by a Creative Commons licence – details of these licences and which Creative Commons licence will apply to this Work are set out in our licence referred to above.

Other than as permitted in any relevant BMJ Author's Self Archiving Policies, I confirm this Work has not been accepted for publication elsewhere, is not being considered for publication elsewhere and does not duplicate material already published. I confirm all authors consent to publication of this Work and authorise the granting of this licence.

For peer review only

'You're just there, alone in your room with your thoughts:' A qualitative study about the psychosocial impact of lockdown among young people during the COVID-19 pandemic

Authors:

Alison. R McKinlay, [a] a.mckinlay@ucl.ac.uk

Tom May, [a] t.may@ucl.ac.uk

Jo Dawes, [a] joanna.dawes@ucl.ac.uk

Daisy Fancourt, [a] d.fancourt@ucl.ac.uk

& Alexandra Burton [a] a.burton@ucl.ac.uk

[a] *Institute of Epidemiology & Health Care, University College London, London, United Kingdom*

Corresponding author: Alison McKinlay, PhD, CPsychol, AFBPsS, University College London, Department of Behavioural Science and Health, Institute of Epidemiology and Health Care, 1-19 Torrington Place, London, WC1E 7HB.

Declaration of interest

None

Funding

The Covid-19 Social Study was funded by the Nuffield Foundation [WEL/FR-000022583], but the views expressed are those of the authors and not necessarily the Foundation. The study was also supported by the MARCH Mental Health Network funded by the Cross-Disciplinary Mental Health Network Plus initiative supported by UK Research and Innovation [ES/S002588/1], and by the Wellcome Trust [221400/Z/20/Z]. DF was funded by the Wellcome Trust [205407/Z/16/Z].

Acknowledgements

The researchers are grateful for the support of the British Youth Music Theatre during recruitment. Many thanks to Louise Baxter, Rana Conway and Sara Esser for their help with conducting interviews. Thank you to Katey Warren and Henry Aughterson who provided feedback on the themes and subthemes. Thank you to the peer reviewers who provided critical feedback and suggestions to improve the manuscript.

Author Contribution Statement

DF and AB designed the study. AB, AM, JD and TM collected data for the study, analysed and interpreted the data. AM wrote the first draft with input from AB, JD and TM. All authors provided critical revisions, read, and approved the submitted manuscript. All authors had full access to the data in the study and can take responsibility for the integrity and accuracy of the data.

Ethical Statement

This research was granted ethical approval by the University College London Ethics Committee (London, Project ID: 14895/005). All participants aged 16-24 provided written informed consent to participate. Participants aged 13-15 provided written assent to participant and a parent also provided their verbal and written informed consent.

Data Availability Statement

For peer review only

The data are not publicly available due to their containing information that could compromise the privacy of research participants.

Abstract

Objectives: Adolescents and young adults have been greatly affected by quarantine measures during the COVID-19 pandemic, but little is understood about how restrictions have affected their wellbeing and social life. We therefore aimed to learn more about how UK quarantine measures affected the social lives, mental health, and wellbeing of adolescents and young adults.

Design: Qualitative interview study. The data were analysed using reflexive thematic analysis, with particular attention paid to contextual factors (such as age, gender, ethnicity, and health status) when analysing each individual transcript.

Setting: Data collection took place remotely across the UK via audio or video call, between June 2020 and January 2021.

Participants: We conducted semi-structured interviews with 37 participants (aged 13-24 years) to elicit their views.

Results: Authors generated four themes during the qualitative analysis: a) concerns about disruption to education, b) missing social contact during lockdown, c) changes to social relationships and d) improved wellbeing during lockdown. Many participants said they struggled with a decline in mental health during the pandemic, lack of support, and concern about socialising after the pandemic. However, some participants described experiences and changes brought on by the pandemic as helpful, including an increased awareness of mental health and feeling more at ease when talking about it, as well as stronger relationship ties with family members.

Conclusions: Findings suggest that young people may have felt more comfortable when talking about their mental health compared to pre-pandemic, in part facilitated by initiatives through schools, universities, and employers. However, many were worried about how the pandemic has affected their education and social connections, and support for young people should be tailored accordingly around some of these concerns.

Keywords – Qualitative Research, Adolescence, Young Adults, COVID-19, Mental Health, Social Support

Strengths and Limitations of this Study

- We interviewed a wide range of adolescents and young adults from across the UK about their experiences during the COVID-19 pandemic.
- Data collection took place during, and in-between several national "lockdown" periods and we elicited their views about the different stages of the pandemic.
- We did not ask participants to provide information about sociodemographic status and it is possible the sample is biased towards those who had the digital and economic means to participate in a remote interview.

For peer review only

Introduction

When coronavirus disease 2019 (COVID-19) was formally declared a pandemic [1] many governments imposed social distancing restrictions to suppress the virus, including social isolation and mobility constraints. Whilst these measures were proposed to minimise contact and virus transmission, there is evidence to suggest that they have resulted in adverse psychological consequences across general populations. [2,3]

Lessons learned from previous epidemics suggest that youth experience measures aimed at preventing the spread of viruses as particularly distressing [4] with the potential for some effects to be long-lasting, even after restrictions have lifted. [5,6] The effect of pandemic-related disruptions in routine may be felt more acutely by children and young people with pre-existing conditions, such as autism spectrum disorder. [7] While youth are less likely to experience severe symptomology or hospitalisation with COVID-19, [8,9] the impact of quarantine represents a significant mental health threat. [10] Emerging evidence suggests that young people have experienced the greatest decline in mental health in the first wave of COVID-19, in comparison to people in other age ranges, [11] and young people identifying as Black and Black British have been most affected in the UK compared to other ethnic groups. [12]

Reasons for this reported decline in mental health among younger people are likely to be multifaceted. Firstly, school closures and education disruptions remove routine, structure and opportunities for socialisation, [13] which may increase loneliness and isolation. For children or young people in need of mental health support, the closing down of schools and universities removed access to coping resources or infrastructures located in these settings (e.g., mental health services and peer support). Secondly, the age span from adolescence to young adulthood is a particularly sensitive one as young people experience major life transitions. Disruption to these transitions can cause uncertainty and anxiety, as documented in young adults experiencing job insecurity resultant from the pandemic [14] or students experiencing the cancellation of their studies. [13] Finally, beyond these individual factors, some adolescents and young adults faced additional stressors during COVID-19, including a decline in parental wellbeing, child maltreatment, and bereavement, all of which may compound changes in circumstances brought on by the pandemic (e.g. online learning, home schooling). [15,16] These experiences were likely felt most acutely by those already affected by socioeconomic marginalisation and deprivation. [17]

In these contexts, young people and adolescents were at risk of unique psychosocial consequences from the pandemic compared to other population groups, and while others have sought to quantify the impact of the pandemic on young people’s mental health, there is limited work exploring young

For peer review only - http://bmjopen.bmj.com/site/about/guidelines.xhtml

people's individual perceptions at various stages of the pandemic. In this research, we sought to explore adolescent and young adult experiences during the COVID-19 pandemic in the UK, specifically to understand how and why social distancing restrictions affected social lives, mental health, and wellbeing.

Method

We undertook a qualitative interview study to explore the experiences of adolescents (aged 13-17) and young adults (aged 18-24) throughout the COVID-19 pandemic in the UK. This work forms part of the COVID-19 Social Study (CSS), which is a national panel survey established in March 2020, where researchers have been investigating the psychosocial wellbeing of people in the UK throughout the pandemic. [18] The research was reviewed and given approval by the University College London Ethics Committee (London, Project ID: 14895/005).

Participant recruitment

Eligibility criteria included: aged 13-24, living in the UK, able to speak English in order to read the study information and to understand and sign a study consent form. All participants aged 16-24 provided written informed consent. Participants aged 13-15 provided assent and a parent provided written informed consent. All participants completed a demographics questionnaire (See Table 1 for participant demographics). No participants were previously known to the interviewers prior to recruitment. In order to make an informed decision about whether to participate, potential participants received a Study Information Sheet with further details on the study, data protection measures and links to organisations providing mental health support. All were given the opportunity to ask questions and no one who was offered an interview declined to take part. No participant response rates were recorded. All participants who participated in interviews were emailed an online £10 shopping voucher as a token of appreciation for their time.

We recruited a convenience sample informed by purposive and snowballing sampling strategies. Our purposive recruitment approach was aligned with "quota sampling," whereby a minimum number of participants in each group (based on age, ethnicity, living situation) were recruited to ensure we were able to learn about the richness of experience across groups. [19] In considering the evidence that certain groups have faced unique challenges during the COVID-19 pandemic (Fancourt et al. 2020), we screened those who registered their interest in attempt to ensure participants were from a range of diverse backgrounds in terms of age, gender, ethnicity, education level, and living situation. The study was advertised via the CSS study newsletter, through partner organisations working with young people, personal contacts and for young adults aged 18+, via social media. Although we screened

For peer review only - http://bmjopen.bmj.com/site/about/guidelines.xhtml

participants to ensure people from multiple groups were included, the nature of our recruitment process meant our sample was ultimately recruited by convenience. [19]

Data collection

In light of the quarantine measures in place at various points throughout data collection, all participant interviews took place remotely, either over telephone or video call. Experienced, postgraduate, male and female, qualitative health researchers conducted all interviews (AB, AM, JD, RC, SE, TM, LB). All interviewers have previously carried out interviews about mental health and social life throughout the COVID-19 pandemic for other CSS research work. [20–22] Interviews followed a semi-structured format (range: 17-65 minutes, average 38), with prompts used to focus on each participant's own lived experience of the COVID-19 pandemic. For adolescents aged 13-17, interviews ranged from 17 to 46 minutes (average: 33 minutes) and for young adults aged 18-24 from 29 to 65 minutes (average: 44 minutes). As the study was designed to elicit experiences of social isolation and social restrictions on mental health, wellbeing and social lives, topic guide questions were informed by social integration and sense of coherence theories. [23,24] The interview topic guide included questions on participants' lives before the pandemic, understanding of and adherence to government restrictions, mental health, and thoughts about the future (for question examples, see Figure 1. For full list, see Supplementary file).

[figure 1 about here]

In order to capture some of the nuanced differences between experiences of adolescents compared with young adults, we made several key choices during data collection and analysis. We conducted data collection as two separate groups according to participant age (group 1: adolescents aged 13-17 years, group 2: young adults aged 18-24). We made this decision in order to focus on experiences that were specific to different stages of development in adolescence and "emerging" adulthood. [25] Where possible in our results, efforts have been made to clarify any similarities and differences in participant responses based on their age group. Rather than progressing in a linear fashion from stage to stage during qualitative data analysis, [26] we made a decision to begin coding before data collection had finished. This approach allowed us to reflect on the way questions were asked during interviews and use prompts to learn more about topics of relevance to the research aims.

Data analysis

Research interviews were the only source of data collected and analysed during the study. We audio recorded interviews, which were transcribed verbatim by a transcription service. Transcripts were double checked for anonymity and accuracy, then imported into NVivo version 12. We did not use

For peer review only - http://bmjopen.bmj.com/site/about/guidelines.xhtml

member checking methods during the study. We incorporated reflexive thematic analysis, [27,28] and used a combination of inductive and deductive coding approaches. First, an initial coding framework was developed based on the theoretical concepts explored in the interview topic guide. This was updated with new codes that were grounded in the data identified whilst reading through the transcripts. AM and TM double coded three interview transcripts in the young adult group and discussed these to ensure coder consistency. AM and JD double coded three transcripts in the adolescent group to ensure consistency across coders in identifying topics of salience. AM and TM then carried out line-by-line coding of interview transcripts and created the coding framework. The list of themes and subthemes were developed iteratively: AM presented themes and subthemes during weekly internal meetings with the CSS research team, in order to gain formative feedback on the comprehensibility of codes and preliminary findings. Findings were revised and finalised based on this feedback.

Patient and public involvement

Patients or members of the public were not involved with the design, conduct, reporting or dissemination of this research.

Results

We recruited 37 participants between June 2020 and January 2021. Participant characteristics are described in Table 1. The average age of participants was 18 (range 13-24 years old). The majority were female (62%) and White British (73%). All participants lived with others (e.g., housemates, family, caregiver, spouse). Most participants were in secondary school or university (84%) and living at home with their parents (78%). Sixteen participants reported that they had an existing physical (7) and/or mental health condition (16).

Table 1 Self-reported demographic characteristics

	Participants n = 37
Age	
13-14	4
15-16	12
17-18	8
19-20	2
21-22	6
23-24	5
Sex	
Female	23
Male	14

For peer review only

Ethnicity	
White British	27
Mixed Race*	5
Asian and Asian British**	4
**Qualifications**	
No qualifications	9
GSCE	9
A Levels	13
Undergraduate	5
Postgraduate	1
**Employment**	
Still at school	17
At university	14
Fulltime employment	3
Apprenticeship	1
Self-employed	1
Unemployed	1

* Participants who identified as Mixed Race also specified that they identify as White and Black African, White and Black Caribbean, White and North African, Other Mixed Background. ** Participants who identified as Asian or Asian British also specified that they identify as Indian, Punjabi, Pakistani, and Malaysian.

We generated four overarching themes about the impact of the pandemic on mental health and wellbeing: a) Concerns about disruption to education, b) Missing social contact during lockdown, c) Changes to social relationships, and d) Improved wellbeing during lockdown. Themes and subthemes are illustrated in Figure 2.

[Figure 2 about here]

Theme One: Concerns about Disruption to Education

The cancellation of assessments and closure of schools and universities were described as a source of loss, concern, and uncertainty for most participants. Many felt unsupported in their transition to online, remote learning and others worried about the longer-term impact of education closures on their future career and learning prospects.

Difficulties with Remote Learning and Increased Workload

Inability to concentrate, feelings of boredom and a lack of stimulation created difficulties with remote learning for some participants.

> *"I was just staring at a take home exam paper with my brain just completely blue screened. You know, just completely not functioning. I think that just general stimulus vacuum of lockdown massively contributed to that because that's an experience I've never had before." P56, male, aged 21-22*

For peer review only - http://bmjopen.bmj.com/site/about/guidelines.xhtml

For those living at home with their families, having to share a workspace with multiple siblings at once, or feeling confined to a bedroom to study because everyone in the household was working from home, meant that "*days felt like night.*"

> *"When I've sat in my room all day, there's no distinguishing between the room where I sleep and the room where I do all my work." P20, male, aged 13-14*

After several months of social distancing and online learning, some participants felt that the move to remote online learning had increased not only their screentime but their workload as well, compared to face-to-face learning.

> *"My school…said we'll do live lessons, but then a teacher, instead of doing live lessons, just put the PowerPoint online, and then we just had to take notes from that. And they'd say, make four pages of notes on this, do the questions and then that's your work done for the lesson, which wasn't very good, because instead of spending one hour a lesson, I'd spend two or three hours." P68, male, aged 17-18*

The impact of this increased workload on future exams and ultimately grades created an additional source of concern and uncertainty among participants.

> *"I know that the virus has definitely had a big toll on how much I'm thinking about [exams], because there's so much uncertainty… My worries about exams [are] more like: 'how am I going to get the content done in time,' not how am I going to be graded." P49, female, aged 15-16*

Uncertainty about Assessments and Exams

Many participants in the sample discussed feeling stressed, particularly at the start of lockdown, when there was a lack of clear guidance over how schools and universities would manage coursework and exams amid education closures.

> *"University was probably biggest stress at the start because there was an uncertain period when we first went into lockdown, where all the universities were rushing to work out what they were going to do. At this point I was looking at handing in a dissertation and sitting three or four exams, and I didn't know if it was still happening." P54, female, aged 20-21*

When announcements were made that exams would be cancelled, many said that they felt upset or disappointed and reported feeling robbed of the chance to complete their education as planned.

> *"I'm actually really upset that I couldn't sit my exams, so yes. It was a bit of an anticlimax, because I kind of wanted all of the build-up and the apprehension, to finalise, what, two years'*

For peer review only - http://bmjopen.bmj.com/site/about/guidelines.xhtml

worth of… Well, my entire education built up to this. And when it was halted… I found it a bit difficult." P59, female, aged 18-19

Some participants in secondary school reported that they had studied hard outside of school hours to improve their grades even before the pandemic hit and were anxious that this additional effort would not be taken into account when considering their grade predictions.

"Not doing exams, I was gutted. I can't prove myself and I'm being given a letter that they think I could get... they didn't see how hard I was working…they don't see how hard we work outside of school… It's just annoying that we can't prove ourselves." P34, female, aged 15-16

Education Expectations did not match Experience

Many participants spoke of feeling disappointment that their social plans for secondary school or university had not materialised due to closures and disruptions. Symbolic events tied to the start or end of education (such as "Freshers week", "Leavers assembly" or prom) were cancelled and described as another source of loss during the pandemic.

"We didn't get the finale of Year 13 like we normally do where there's like a prom and things like that and it was all just a bit rushed so we didn't get to say bye." P53, male, aged 18-19

Some said that they felt they had "missed out" on an experience that they saw as a rite of passage and one that they had been looking forward to.

"Because we were planning on going out so much and that all being cancelled, it was awful. The day when I was meant to have prom I put my prom dress on just because I wanted to… it was like the idea of it all being cancelled I felt like I was missing out." P55, female, aged 15-16

Lack of Support and Communication

Many participants described feeling that there was a lack of support for their mental health and wellbeing from education providers during the pandemic.

"The school hasn't been great in terms of providing help… the general attitude is: 'it's okay not to be okay, but we're not going to do anything about it.' I know a couple of other schools have a similar situation where you feel like, do they know that you're struggling, but they're not going to do anything." P61, female, aged 15-16

In cases where support was offered, some said that they had experienced this as performative rather than genuine.

For peer review only - http://bmjopen.bmj.com/site/about/guidelines.xhtml

> *"And I feel really let down by the school…Our head of year did a wellbeing check and it didn't even seem genuine, it just seemed like she had to do it for her ego or something like that..." P34, female, aged 15-16*

Others described poorly managed communication and a lack of information from education providers about pandemic-related changes, which left students uncertain about their options for rearranging assessments and coursework.

> *"It would have been nice if I could speak to my personal tutor or something about it, but I think everyone was so busy at the beginning trying to record lectures and put them online, and everything was a bit hectic. So, if there was support it wasn't very clear or easy to find," P64, female, aged 20-21*

Poor communication and a perceived lack of support from education providers also resulted in some students feeling forgotten about or unfairly treated. This led to feelings of anger, frustration and a sense that their experiences were not valued.

> *"At the beginning, kind of March to May period, the response was absolutely shambolic and pretty malevolent to be honest…telling people to go home at their own expense up to and including sort of international students being told that they had to leave within a week even if that meant that their flights were two grand or something." P56, male, aged 20-21*

Theme Two: Missing Social Contact During Lockdown

Many participants reported a decline in wellbeing due to feeling lonely and isolated, particularly at the start of lockdown. Being unable to see friends or family and feeling that they were missing out on important life events was a source of sadness and frustration. Some withdrew themselves even further from online social activities after government restrictions were imposed.

Struggling with Isolation

Most participants said that their mental health was affected by social distancing restrictions. Many described this as being due to feeling socially restricted or "trapped" at home and being unable to maintain their usual activities and relationships.

> *"I'm not a very anxious person. I've struggled with mental health anyway, but… I think in the beginning of lockdown, being trapped at home wasn't ideal in terms of my mental health." P59, female, aged 18-19*

For peer review only - http://bmjopen.bmj.com/site/about/guidelines.xhtml

The feeling of being inside and at home for an indeterminate period of time was overwhelming for some, leading to a desire to overcompensate for what was perceived as "lost time" when restrictions began to ease after the first wave of the virus.

> *"As soon as restrictions were relaxed enough that you could meet someone socially distanced, I was down at my friends' houses that are 100m down the road, three or four times a week, just to get out of my house. I got so frustrated being cooped up." P55, female, aged 15-16*

The social distancing restrictions were especially challenging for participants who had prior mental health concerns, many of whom reported that their wellbeing had declined while they were socially isolating.

> *"…during lockdown, I got horrendously depressed. So, I've had stuff going on mentally, but I was finding it tough. The social isolation made everything crash really badly." P39, male, aged 21-22*

Social Withdrawal and Loneliness

While many participants had adapted their lives in response to social distancing restrictions, some, particularly adolescents, described socialising less during the lockdown and reported feelings of loneliness or social withdrawal.

> *"I don't really speak to much of my mates as I used to. It's made me quite distanced with quite a lot of people." P66, female, aged 16-17*

At the start of the lockdown, some participants felt uncertain or overwhelmed by the move to online social interactions and made a conscious decision to withdraw from this method of contact.

> *"Before lockdown, I never used video calls or phoning people anyway. I spoke to people through messaging a lot, but I never used Zoom. I never heard of it to be honest. So, when I was suddenly forced to do that, it was just too much, so I've stopped talking to people." P20, male, aged 13-14*

Boredom was commonly reported by a majority of participants and lowered their motivation to interact with others. As the pandemic went on, some participants said that they had nothing to talk about with their peers, or that friends were uninterested in keeping in contact. Therefore, efforts to maintain social connection ceased and usual social ties further disrupted.

> *"I definitely tried to keep in touch with my school friends more at the start, but then it felt very one-sided, so I stopped making all the effort and I haven't spoken to a few of them in a few months." P44, female, aged 17-18*

For peer review only - http://bmjopen.bmj.com/site/about/guidelines.xhtml

For peer review only

Participants described feeling lonely throughout different stages of the pandemic, as though the world felt smaller from being at home during the lockdown without any social contact.

> *"There was some socialising over Zoom but overall, I think my world just sort of contracted fairly significantly…" P56, male, aged 21-22*

Several participants felt lonely even in the presence of others as restrictions began to ease (but rules such as mask-wearing and no touching remained in place), with many associating their feelings of loneliness with a lack of physical contact and closeness.

> *"So, even though we are back at school, we're still struggling through the same things. We're still feeling lonely, that we're not able to have that physical contact, which I'm looking forward to having if the pandemic gets better." P51, female, aged 15-16*

Theme Three: Changes to Social Relationships

Relationship Tensions

Participants spoke of stress, anxiety and anger regarding family and friendship tensions during the pandemic. For some, this was due to not being permitted to see their friends, which led to relationship breakdowns.

> *"What's changed is it's put a lot of pressure on relationships, like with my boyfriend and a lot of people I know of have broken up with their boyfriends or girlfriends because of this quarantine and not being able to see them every day, or be around them as much." P19, female, aged 15-16*

For others, differences in opinions between friends led to arguments over what was acceptable or not when following the social distancing rules.

> *"One of my mates, my mate, I actually ended up having an argument, because she was, like, oh, you've got to come and see me." P66, female, aged 17-18*

Participants living with family members said that they experienced difficulties getting along with parents and siblings during lockdown, particularly when living together in small homes or when everyone was working from home.

> *"I don't always get on with my family very well and we would be in each other's faces a hell of a lot and that got very difficult at times… I'd say it gets worse when we have to spend more time together." P38, male, aged 18-19*

For peer review only - http://bmjopen.bmj.com/site/about/guidelines.xhtml

"I think everyone just got a little bit stir crazy seeing the same walls and the same people." P44, female, aged 17-18

Relationship Unity and Connectedness

Despite relationship tensions, participants also described feeling closer with their family members throughout the pandemic and specifically during lockdown periods spent together.

"My relationship with my parents is great and it's improved. It was at a great point this year and I think better than it's ever been. And I think lockdown was a good time just to spend more time with them." P37, female, aged 23-24

Many were reminded about the importance of family and became resolute to make more effort to connect with others in the future.

"I won't take for granted as much, like just standing outside talking to my neighbour, phone calls with my [relative]. Just the little things like that. Because obviously it's so easy in normal life just to brush them aside and say, 'oh, I'll ring my [relative] later.' So now I take the time to ring my [relative] and talk to her." P33, female, aged 1516

Some participants had "difficult" conversations with family during the lockdown that they otherwise might not have had, for instance, what they would do if a family member became ill with the virus. Having frank and open conversations helped some to feel more connected with their family members than before the pandemic.

"I definitely think [the pandemic] forced us to talk about things that we wouldn't always want to talk about. We had to discuss as a family what would happen to my dad if he was to get ill…We discussed those things already in general because we are quite open about that but I think it increased the intensity and frequency of those conversations about death and what would happen and health and things like that… it's helped us to be more open with each other" P41, female, aged 23-24

Adapting to Maintain Social Connections

In response to social distancing measures, participants described the ways in which they had adapted their social lives during the pandemic, including trying new methods of online communication.

"The idea of not knowing when you're next going to be in a crowded place, really able one on one to chat to people I think has just made people search for other ways of connecting with people and that's online." P41, female, aged 23-24

For peer review only

Some said they were using dating apps, chatting through gaming platforms and sharing COVID-related "memes" as a way "to cope with difficult things" and enhance their sense of connectedness with others. Older participants in particular said that they were more focused on online dating during lockdown as a way to connect: *"I joined a dating app just to meet people."* And others sought out dating apps as a way of alleviating boredom.

> *"I personally don't think I would've downloaded that [dating] app if it hadn't have been for the pandemic because I've tried them before and really didn't like them and hadn't had much success. And then I was just bored and felt like when am I next going to meet someone?... I feel like that probably wouldn't have happened if it wasn't for lockdown." P77, female, aged 23-24*

New friendships were formed during lockdown with the help of chat functions on gaming and social media platforms (i.e., "Whatsapp").

> *"I have made a bunch of friends that are from online around my age from playing a game...so during the pandemic instead of chatting with my friends I'd be playing a game with them almost every day. Well, every day for a few hours chatting, having fun which was nice." P21, male, aged 15-16*

Changes in Feelings about Socialising after Lockdown

Around half of participants described how their feelings about in-person social interactions had changed since the start of the pandemic. Some described this in terms of feeling more vigilant about being around other people and of the risks associated with future virus transmission.

> *"I think I'm going to be a lot more cautious about other people, obviously, like people you don't know being quite close to you, physically." P45, male, aged 15-16*

Although individual concern about their own risk of catching COVID was not widespread in the group overall, some participants still reported feeling safer during lockdown away from other people and less exposed to the perceived risk of catching COVID-19.

> *"I felt less anxious about everything when we were in full lockdown and that the lifting out of lockdown, that's quite scary in many ways for many different people…obviously introducing more risk into your life, whereas when everyone was in full lockdown, I feel like there was something quite comforting in a weird way about that." P37, female, aged 23-24*

Adolescents in particular said they felt apprehensive about future social situations from not having been exposed to "*real life*" and becoming accustomed to life in lockdown.

For peer review only - http://bmjopen.bmj.com/site/about/guidelines.xhtml

"I just feel like I've lost the ability to form coherent sentences. I know that sounds really stupid. It's just been really hard." P34, female, aged 15-16

Concern about having to socialise was especially present in those returning to secondary school after months in lockdown, following difficulties in keeping in touch with friends and maintaining positive friendships.

"I was nervous, because a lot changed during quarantine. I think people got really tetchy staying at home, so there were a lot of arguments. Loads of people fell out of contact with one another…So, it was harder for us to find common things to talk about, we were just speaking about the same topics again and again. So, going back [to school, I] was really nervous because we didn't know what we would talk about." P49, female, aged 15-16

Theme Four: Improved Wellbeing During Lockdown

For those who experienced busy and stressful lives prior to the pandemic, social distancing restrictions provided a chance to pause and reflect, with resultant mental health benefits. Having more choice about how to spend free time was described by participants as relaxing and removed previous sources of stress. For some, their wellbeing was enhanced by support from others and an increased public awareness of mental health self-management during lockdown.

Improved Mental Health Compared to Pre-pandemic

In comparison to a majority of participants who reported struggling with their wellbeing during the pandemic, a smaller group of adolescents described how their mental health had improved during the first lockdown.

"Well, before lockdown, my mental health wasn't great… my sleep schedule was all over the place, school stress, but actually, lockdown gave me a chance to focus and make myself a lot better in terms of the positive emotions" P49, female, aged 15-16

They described busy or stressful school lives prior to the pandemic. School closures and a shift to online learning, particularly for those who were not preparing for their exams, meant that some had less work to do. Social distancing restrictions and pandemic-related closures therefore provided an opportunity to relax and reflect.

"I think it's been just I guess inconvenience rather than a full crisis in my eyes. It probably should have affected me more but, yes, I quite like it in quarantine to be honest. I don't have to get up, I can just chill around. It's a lot nicer than having to rush around constantly." P21, male, aged 15-16

For peer review only

For peer review only - http://bmjopen.bmj.com/site/about/guidelines.xhtml

As previously reported, the closure of schools and universities meant an increased workload for some, but the flexibility of choosing what to do between lessons and during free time meant the lockdown felt "*like a really long summer holiday*" for others.

> *"I was happy when school got cancelled… it was still hard to do the work at home, but it was just because it was just like, in between I could just do whatever I wanted, so it was just better, sometimes I just go and play in the garden, football, or just be on my phone or the PS4." P28, male, aged 13-14*

Increased Self-Awareness

Throughout the course of the pandemic, some participants had more time to take up hobbies and exercise, but several young adults also commented on the personal growth and increased self-awareness that they had observed.

> *"[This experience has] taught me to take everything a day as it comes. And then see what happens. But I won't be surprised if, in a good way, that things change and it happens for the better.… it allowed me to challenge myself more... now things have started to flow better with how I am as a person." P60, female, aged 21-22*

Some participants reflected on the stark changes in their lives during the pandemic compared with pre-pandemic and said they felt differently about how they wanted their life to be when social distancing restrictions eased.

> *"First, I think someone hit the brakes, I think, like everything stopped…[Life is] no longer that constant, hectic mess of going out…overall, it's a lot less intense maybe, now." P39, male, 21-22*

Increased Awareness of Mental Health

Some participants said they felt an increased awareness of their own mental health and how to protect it during the pandemic, through initiatives driven by workplaces and education providers.

> *"My organisation did allocate three weeks of wellbeing leave which is a really interesting move. And that was on top of annual leave. And that was complementary leave because the reason why they did it is that they said that it's a really hard time and they just think everyone needs the break." P37, female, aged 23-24*

Some said they had not paid attention to their mental health in the past compared to now, having been through a pandemic and enforced social isolation.

For peer review only - http://bmjopen.bmj.com/site/about/guidelines.xhtml

“[the pandemic has] shown that health and mental health and mental state is important. As it always was, but I don’t think I realised it as much as I would now.” P46, male, aged 15-16

This perceived change in mental health awareness helped make it feel easier to talk to others about their wellbeing, and for some, this “opened up” a dialogue with friends and family that had never been achieved before.

“It opened up really big conversations within our family about mental health that I don't think they’ve ever had before like the generations. So those are two really big positives for me I think. Both myself and opening that up wider...It was just conversations that we’d never had before.” P37, female, aged 23-24

Accessing Mental Health Support

Some participants sought support to help cope with pandemic-related distress, either informally through friends and family, or formally through education providers, health services, or employers.

“I did talk to one of my teachers actually at school, on Teams…And she helped me and a lot of friends about things to do during lockdown.” P50, female, aged 15-16

In some cases, regular offers of support and check-ins by educational staff were sufficiently reassuring to know that help was on hand if needed.

“[University staff would] email every week, checking in. They gave me two… deadline extensions, for my work, which helped me a lot. And my tutor was really helpful. He did check in every couple of weeks to make sure I was all right. Even over the summer, when they haven’t necessarily been at uni. So, that was good.” P57, female, aged 21-22

Discussion

We sought the views of adolescents and young adults about their wellbeing and social lives during the COVID-19 pandemic using detailed qualitative interviews. Young people described mental health consequences associated with education closures and lockdown measures, including loneliness, frustration, a sense of loss about cancelled plans and assessments, and uncertainty about socialising in the future. Our findings highlight how some felt paradoxically isolated but also hesitant about reintegrating with others as the lockdown restrictions ease. For some, the pandemic and associated restrictions provided a platform for new relationships to develop and existing relationships to strengthen, while for others, social isolation added strain and divisions. Despite the challenges, we found evidence of a potentially positive change during the pandemic, including an increased

For peer review only - http://bmjopen.bmj.com/site/about/guidelines.xhtml

awareness of mental health self-management, and having more open conversations about mental health compared to pre-pandemic.

While the long-term effect of school closures on young people's health, income and productivity is yet to be fully realised, [29] disruptions to education on this scale have not occurred since the second World War. [30] These changes will likely have long term consequences requiring ongoing support, [31] with particular attention needed on how sociodemographic factors have affected young peoples' experiences during the pandemic. [32] Around 60% of students living in affluent areas had access to online learning during the pandemic, compared with 23% of students living in more deprived areas. [33] A loss of learning has already been observed by teaching staff in the UK, [34] and our findings suggest this may be linked to increased workloads, sharing small workspaces at home, and difficulties with concentration. If unaddressed, this may restrict opportunities for employment and social mobility available to young people in the future. [31] It is essential that those without the resources to work optimally from home are supported, to ensure that educational and social inequalities are not exacerbated, both as COVID-19 persists and in other future scenarios where remote learning may be required.

It is estimated more than half of students have experienced adverse psychological wellbeing during the COVID-19 pandemic. [35] We found that paradoxically, while some participants felt isolated and lonely, they were also apprehensive about social distancing restrictions easing and life returning to "normal". Graded or phased approaches for those returning to education [36] and employment are recommended as lockdown restrictions ease. [37] Academic challenges have been frequently reported by young people during the pandemic, particularly among secondary school aged youth. [32] We also noted concern among those who had access to online learning but were unable to sit exams and finish their coursework. Several young people felt they did not demonstrate their full potential when their exams were cancelled and did not want to be known as the generation who were given a "free pass". Scott et al. also found young people reported feeling less confident and like an "imposter" following cancelled exams due to the pandemic. [38] Teaching staff may be well-placed to address these concerns by clearly communicating revised assessment plans and providing reassurance about the assessment process for future students affected by pandemic-related education disruptions. Teachers also require clear guidance and coordination from government and educational authorities to be able to deliver these messages, [34] which were absent during the first wave of educational establishment closures.

Young people have reported some of the highest rates of loneliness during the pandemic, [39] which have been associated with a decline in psychological wellbeing. [40] Initiatives that enable young

For peer review only - http://bmjopen.bmj.com/site/about/guidelines.xhtml

people to easily connect with others and access support for their mental health while adhering to social distancing guidelines are greatly needed. [40] Participants in our study frequently cited mental health consequences due to social isolation and loneliness, which some used gaming platforms, social media, and online dating apps to help address. These findings build on those shown in previous research involving young people during the first national lockdown, [41] including the sense of loss and uncertainty frequently reported by this group. Our results build on this by suggesting how social isolation led to these effects, including what felt like missed opportunities to create memories with friends and missed social events seen as rites of passage in young adulthood.

The effects of isolation and loneliness for young people are likely to persist for some time, even after social distancing restrictions have lifted. [42] The path ahead will require additional resources for education providers and coordinated efforts from policy makers, education authorities, and health and social care to provide reassurance that service provision is responsive to the needs of young people. [43] This response should be formulated in collaboration with young people to ensure that any future support incorporates appropriate coping strategies and protective activities, [44] such as those identified by our study, including check-ins from education providers and wellbeing initiatives from employers. These responses could help to validate the unique struggles that many young people have faced throughout the different stages of the pandemic. [44]

Despite the many negative effects of the pandemic on mental health in young people identified to date, evidence also suggests that young people's altruistic motivations [45,46] and prosocial behaviours have increased during the pandemic, including providing practical support to others and donating to charities perceived to be in need. [47] It is encouraging that some participants in this study reported feeling more able to talk about their mental health compared with pre-pandemic, suggesting less social stigma perceived by young people in this study to be associated with mental health and wellbeing. This was, at times, facilitated by top-down efforts from education providers, employers, and parents. In this study and others, [20] we found that many have been reminded of the importance of family and friends during adversity. These changes represent a potentially positive side effect of the pandemic, providing they are supported and maintained.

Strengths and Limitations

To our knowledge, this is the first UK study to use qualitative interviewing methods to learn more about the mental health and wellbeing of young people (aged 13-24) during multiple phases of the COVID-19 pandemic. Purposive recruitment strategies were used to try and increase the variability of experiences within the group of participants interviewed, but despite this, our sample contained more 15- and 16-year-olds than other age groups, and participants who identified as White than other

For peer review only - http://bmjopen.bmj.com/site/about/guidelines.xhtml

ethnic groups. Those whom we spoke with were from a range of living situations and health statuses; however, there were some data we did not collect that is likely to have influenced participant experiences during the pandemic, including socioeconomic status, sexual orientation, and gender expression. Data were gathered throughout several key time points of the UK pandemic response, including as schools were reopening after the first lockdown, and as the second wave of the virus had begun. We explored a range of issues during interviews that were pertinent to the research questions, but some topics identified during this study could be investigated in further depth by future researchers, including the impact of the COVID-19 pandemic on intimate relationships among adolescents and young adults. We relied on participants self-identifying to take part in the research, which may have meant that the sample whose experiences were more positive than those less comfortable discussing their experiences or with less time to take part in research. Remote interviewing techniques may have excluded some groups with limited online access; however, we also offered to conduct telephone calls and remote data collection methods enabled us to speak to people from across the UK.

Conclusions

When the pandemic began and restrictions were introduced, many young people lost a sense of certainty, community and belonging provided by their routines, social circles, and education providers. Our findings highlight how support for adolescents and young people to develop and maintain their social connections and access mental health support is needed even after the virus is controlled. Findings suggest that young people in the study may have felt less stigma talking about their mental health now compared to pre-pandemic, which was in part facilitated by initiatives through schools, universities, and employers. Most participants in our study struggled with their wellbeing and loneliness during the pandemic, and our results highlight the vital role of education providers giving regular guidance and support to help alleviate their concerns. Many young people we spoke with are worried about how the pandemic has affected their education and social connections, and support for young people should be tailored accordingly around these concerns. We also highlight the detrimental impact that an absence of information and support had on young people's mental health. More must be done to ensure offers of support to young are genuine and reinforced with signposting to information, advice and services when young people do reach out for help.

For peer review only - http://bmjopen.bmj.com/site/about/guidelines.xhtml

Figure captions

1. Figure 1: Interview guide examples
2. Figure 2: Themes and sub-themes

References

1. World Health Organisation. Coronavirus disease 2019 (COVID-19) Situation Report – 51 [Internet]. 2020. Available from: https://www.who.int/docs/default-source/coronaviruse/situation-reports/20200311-sitrep-51-covid-19.pdf?sfvrsn=1ba62e57_10

2. Niedzwiedz CL, Green MJ, Benzeval M, Campbell D, Craig P, Demou E, et al. Mental health and health behaviours before and during the initial phase of the COVID-19 lockdown: longitudinal analyses of the UK Household Longitudinal Study. J Epidemiol Community Health. 2020 Sep 25;jech-2020-215060.

3. Pierce M, Hope H, Ford T, Hatch S, Hotopf M, John A, et al. Mental health before and during the COVID-19 pandemic: a longitudinal probability sample survey of the UK population. Lancet Psychiatry. 2020 Oct;7(10):883–92.

4. Sprang G, Silman M. Posttraumatic Stress Disorder in Parents and Youth After Health-Related Disasters. Disaster Med Public Health Prep. 2013 Feb;7(1):105–10.

5. Brooks SK, Webster RK, Smith LE, Woodland L, Wessely S, Greenberg N, et al. The psychological impact of quarantine and how to reduce it: rapid review of the evidence. The Lancet. 2020 Mar;395(10227):912–20.

6. Kwong ASF, Pearson RM, Smith D, Northstone K, Lawlor DA, Timpson NJ. Longitudinal evidence for persistent anxiety in young adults through COVID-19 restrictions. Wellcome Open Res. 2020 Aug 19;5:195.

7. O'Sullivan K, Clark S, McGrane A, Rock N, Burke L, Boyle N, et al. A Qualitative Study of Child and Adolescent Mental Health during the COVID-19 Pandemic in Ireland. Int J Environ Res Public Health. 2021 Jan 25;18(3):1062.

8. Bhopal SS, Bagaria J, Olabi B, Bhopal R. Children and young people remain at low risk of COVID-19 mortality. Lancet Child Adolesc Health. 2021 Mar 11;

9. Ludvigsson JF. Systematic review of COVID-19 in children shows milder cases and a better prognosis than adults. Acta Paediatr. 2020 Jun;109(6):1088–95.

10. Fegert JM, Vitiello B, Plener PL, Clemens V. Challenges and burden of the Coronavirus 2019 (COVID-19) pandemic for child and adolescent mental health: a narrative review to highlight clinical and research needs in the acute phase and the long return to normality. Child Adolesc Psychiatry Ment Health. 2020 Dec;14(1):20.

11. Fancourt D, Steptoe A, Bu F. Trajectories of anxiety and depressive symptoms during enforced isolation due to COVID-19 in England: a longitudinal observational study. Lancet Psychiatry

For peer review only - http://bmjopen.bmj.com/site/about/guidelines.xhtml

[Internet]. 2020 Dec [cited 2020 Dec 11]; Available from: https://linkinghub.elsevier.com/retrieve/pii/S221503662030482X

12. Dewa LH, Crandell C, Choong E, Jaques J, Bottle A, Kilkenny C, et al. CCopeY: A Mixed-Methods Coproduced Study on the Mental Health Status and Coping Strategies of Young People During COVID-19 UK Lockdown. J Adolesc Health. 2021 Apr;68(4):666–75.

13. Lee J. Mental health effects of school closures during COVID-19. Lancet Child Adolesc Health. 2020 Jun;4(6):421.

14. Ganson KT, Tsai AC, Weiser SD, Benabou SE, Nagata JM. Job Insecurity and Symptoms of Anxiety and Depression Among U.S. Young Adults During COVID-19. J Adolesc Health. 2021 Jan;68(1):53–6.

15. Romanou E, Belton E. Isolated and struggling: Social isolation and the risk of child maltreatment, in lockdown and beyond. [Internet]. 2020. Available from: https://learning.nspcc.org.uk/media/2246/isolated-and-struggling-social-isolation-risk-child-maltreatment-lockdown-and-beyond.pdf

16. Young Minds. Coronavirus: Impact on young people with mental health needs. [Internet]. 2021. Available from: https://youngminds.org.uk/media/4350/coronavirus-report-winter.pdf

17. Waite P, Pearcey S, Shum A, Raw J, Patalay P, Creswell C. How did the mental health of children and adolescents change during early lockdown during the COVID-19 pandemic in the UK? [Internet]. PsyArXiv; 2020 Dec [cited 2021 Feb 26]. Available from: https://osf.io/t8rfx

18. Bu F, Steptoe A, Mak HW, Fancourt D. Time-use and mental health during the COVID-19 pandemic: a panel analysis of 55,204 adults followed across 11 weeks of lockdown in the UK. 2020; Available from: https://www.medrxiv.org/content/10.1101/2020.08.18.20177345v1

19. Campbell S, Greenwood M, Prior S, Shearer T, Walkem K, Young S, et al. Purposive sampling: complex or simple? Research case examples. J Res Nurs. 2020 Dec;25(8):652–61.

20. Aughterson H, McKinlay AR, Fancourt D, Burton A. Psychosocial impact on frontline health and social care professionals in the UK during the COVID-19 pandemic: a qualitative interview study. BMJ Open. 2021 Feb 1;11(2):e047353.

21. Burton A, McKinlay AR, Aughterson H, Fancourt D. Impact of the Covid-19 pandemic on the mental health and wellbeing of adults with mental health conditions in the UK: A qualitative interview study. Medrxiv Prepr Serv Health Sci [Internet]. 2020; Available from: https://www.medrxiv.org/content/10.1101/2020.12.01.20241067v1

22. May T, Aughterson H, Fancourt D, Burton A. 'Stressed, uncomfortable, vulnerable, neglected': a qualitative study of the psychological and social impact of the COVID-19 pandemic on UK frontline keyworkers. SocArXiv [Internet]. 2021; Available from: https://osf.io/preprints/socarxiv/dn43c/

23. Antonovsky A. Health, stress, and coping. San Francisco, US: Jossey-Bass; 1979.

24. Berkman LF, Glass T, Brissette I, Seeman TE. From social integration to health: Durkheim in the new millennium. Soc Sci Med. 2000 Sep;51(6):843–57.

For peer review only

For peer review only - http://bmjopen.bmj.com/site/about/guidelines.xhtml

25. Arnett JJ. Emerging adulthood: A theory of development from the late teens through the twenties. Am Psychol. 2000;55(5):469–80.

26. Elliott V. Thinking about the Coding Process in Qualitative Data Analysis. Qual Rep [Internet]. 2018 Nov 24 [cited 2021 Sep 30]; Available from: https://nsuworks.nova.edu/tqr/vol23/iss11/14/

27. Braun V, Clarke V. Reflecting on reflexive thematic analysis. Qual Res Sport Exerc Health. 2019 Aug 8;11(4):589–97.

28. Braun V, Clarke V. One size fits all? What counts as quality practice in (reflexive) thematic analysis? Qual Res Psychol. 2020 Aug 12;1–25.

29. Viner RM, Russell SJ, Croker H, Packer J, Ward J, Stansfield C, et al. School closure and management practices during coronavirus outbreaks including COVID-19: a rapid systematic review. Lancet Child Adolesc Health. 2020 May;4(5):397–404.

30. d'Orville H. COVID-19 causes unprecedented educational disruption: Is there a road towards a new normal? PROSPECTS. 2020 Oct;49(1–2):11–5.

31. The British Academy. THE COVID DECADE Understanding the long-term societal impacts of COVID-19 [Internet]. 2021. Available from: https://www.thebritishacademy.ac.uk/documents/3238/COVID-decade-understanding-long-term-societal-impacts-COVID-19.pdf

32. Scott SR, Rivera KM, Rushing E, Manczak EM, Rozek CS, Doom JR. "I Hate This": A Qualitative Analysis of Adolescents' Self-Reported Challenges During the COVID-19 Pandemic. J Adolesc Health. 2021 Feb;68(2):262–9.

33. Cullinane C, Montacute R. COVID-19 and Social Mobility Impact Brief #1: School Closures [Internet]. 2020. Available from: https://www.suttontrust.com/wp-content/uploads/2021/01/School-Shutdown-Covid-19.pdf

34. Kim L, Leary R, Asbury K. 'We need clear directions, if we're going to move forward. It's as simple as that': Teachers' narratives during partial school reopenings in the COVID-19 pandemic. PsyArXiv. 2021;

35. Office for National Statistics. Coronavirus and the impact on students in higher education in England: September to December 2020 [Internet]. 2020. Available from: https://www.ons.gov.uk/peoplepopulationandcommunity/educationandchildcare/articles/coronavirusandtheimpactonstudentsinhighereducationinenglandseptembertodecember2020/2020-12-21

36. Viner RM, Bonell C, Drake L, Jourdan D, Davies N, Baltag V, et al. Reopening schools during the COVID-19 pandemic: governments must balance the uncertainty and risks of reopening schools against the clear harms associated with prolonged closure. Arch Dis Child. 2021 Feb;106(2):111–3.

37. Habersaat KB, Betsch C, Danchin M, Sunstein CR, Böhm R, Falk A, et al. Ten considerations for effectively managing the COVID-19 transition. Nat Hum Behav. 2020 Jul;4(7):677–87.

For peer review only

38. Scott S, McGowan VJ, Visram S. 'I'm Gonna Tell You about How Mrs Rona Has Affected Me'. Exploring Young People's Experiences of the COVID-19 Pandemic in North East England: A Qualitative Diary-Based Study. Int J Environ Res Public Health. 2021 Apr 6;18(7):3837.

39. Bu F, Steptoe A, Fancourt D. Loneliness during a strict lockdown: Trajectories and predictors during the COVID-19 pandemic in 38,217 United Kingdom adults. Soc Sci Med. 2020 Nov;113521.

40. Lee C, Cadigan C, Rhew I. Increases in Loneliness Among Young Adults During the COVID-19 Pandemic and Association With Increases in Mental Health Problems. J Adolesc Health. 2020;

41. Demkowicz O, Ashworth E, O'Neill A, Hanley T, Pert K. Teenagers' Experiences of Life in Lockdown: The TELL study briefing. 2020; Available from: https://www.seed.manchester.ac.uk/education/research/impact/teenagers-experiences-of-life-in-lockdown/

42. Loades ME, Chatburn E, Higson-Sweeney N, Reynolds S, Shafran R, Brigden A, et al. Rapid Systematic Review: The Impact of Social Isolation and Loneliness on the Mental Health of Children and Adolescents in the Context of COVID-19. J Am Acad Child Adolesc Psychiatry. 2020 Nov;59(11):1218-1239.e3.

43. Kim LE, Asbury K. 'Like a rug had been pulled from under you': The impact of COVID-19 on teachers in England during the first six weeks of the UK lockdown. Br J Educ Psychol. 2020 Dec;90(4):1062–83.

44. Efuribe C, Barre-Hemingway M, Vaghefi E, Suleiman AB. Coping With the COVID-19 Crisis: A Call for Youth Engagement and the Inclusion of Young People in Matters That Affect Their Lives. J Adolesc Health. 2020 Jul;67(1):16–7.

45. August R, Dapkewicz A. Benefit finding in the COVID-19 pandemic: College students' positive coping strategies. J Posit Sch Psychol. 2020 Dec 8;5(2):73–86.

46. Fisher H, Lambert H, Hickman M, Yardley L, Audrey S. Experiences of the coronavirus disease-19 (COVID-19) pandemic from the perspectives of young people: Rapid qualitative study. Public Health Pract. 2021 Nov;2:100162.

47. Alvis L, Douglas R, Shook N, Oosterhoff B. Associations between Adolescents' Prosocial Experiences and Mental Health During the COVID-19 Pandemic. PsyArXiv [Internet]. 2020; Available from: https://psyarxiv.com/2s73n

For peer review only

For peer review only - http://bmjopen.bmj.com/site/about/guidelines.xhtml

Figure 1 Interview Guide Examples

- *How do you feel about the changes that have been brought about by Covid-19?*
- *Have they had any impact on your mental health or wellbeing?*
- *What are the things most bothering you at the moment?*
- *Has the pandemic meant that you have any worries for the future?*

209x297mm (300 x 300 DPI)

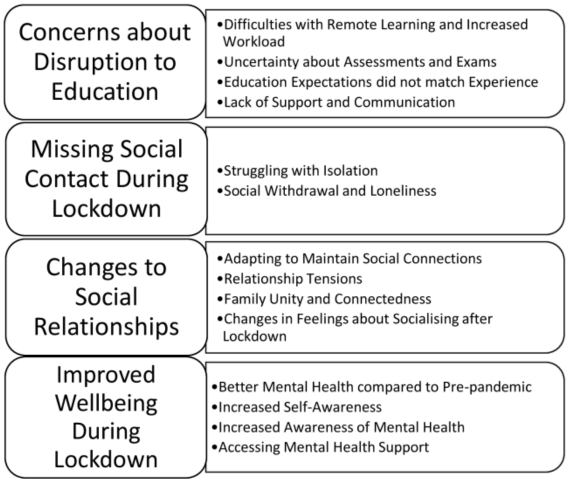

Figure 2 Themes and sub-themes

209x297mm (300 x 300 DPI)

Supplementary Material 1: Topic Guide

Please could you describe what normal life was like for you before the pandemic and the social distancing? (can explain before – e.g. staying at home, stopping visiting people's houses, having to stand 2 metres apart, etc)

- Who do you normally live with separated/ extended family?
- Do you have any siblings/ pets?
- School type, do you have friends you would normally visit or would visit you – please tell me about this?
- Do you have any hobbies or things you enjoyed doing out of school time?

UNDERSTANDING AND ADHERENCE TO GUIDELINES

At the moment, are you 'staying at home'?

- Can you tell me what this is like for you and your family?
    - i.e. are you/ your parents going out for shopping or for walk/ exercise
    - Or staying inside with no outside exercise?/ other people dropping off shopping?

- **What do you understand by the 'social distancing' advice that is being given – what does it mean to you? (Ask to list the rules they are aware of)**
- Have you been…
    - Avoiding crowds
    - Keeping distance from others
    - Isolating (staying inside)
    - Avoiding close contact greetings
    - Socialising/going out only with those in your household

Have you been able to stick to the social distancing advice that has been given? Please tell us about why/ why not?

- What helps you to stick to it?
- Or – what are you finding difficult about sticking to the rules?
- What has motivated you to do this?
- Is there anything that makes it easier to do or harder

[COM-B prompts can be used here, to include:]

- Any existing physical or mental health problems
- Group membership/ applicability Whether it feels like the rules apply to you
- Beliefs about whether it will keep you healthy
- Beliefs about whether it will keep other people healthy
- Having to go out for things like food
- Work/ Caring responsibilities, providing emotional support
- Friends suggesting meeting, family or parents wanting to go out
- Government rules/punishments,
- Feelings about losing normal life
- Change of routine/ habits

For peer review only

For peer review only - http://bmjopen.bmj.com/site/about/guidelines.xhtml

SOCIAL LIFE – In terms of before the pandemic, How would you describe your social life before the Covid-19 pandemic started?
Although some of the question seems less directly relevant, we are trying to understand a big picture of how groups affect/ influence how we feel.

- How would you describe the group of people you knew before the pandemic (these may be the same as now)
  - Are they friends or family or both mainly?
  - Do they live near you or far away?
  - How often did you see each other?
- Did you speak to family and friends face to face/online?

- Were you involved in any groups outside school – please describe (examples such as choir, dancing classes, football teams, youth groups/ youth theatre, Scouts/Guides, social activities etc)
- Did you have people who helped you, such as teachers, Scout leaders, parents, grandparents, family friends? How did they help you, for example listening, help with homework, help with mental health?
- Can you tell us about whether or not your friendship groups encouraged you to get involved in things? Did you find you compared your life to theirs?
- Social roles possibly as a young carer/ older sibling

How would you describe your social life now because of Covid-19?

Please tell us about this – make clear that we are talking about after the start of the pandemic, to see if anything has changed.

Prompts include:

- How would you describe the group of people you know now?
  - Are they friends or family or both mainly?
  - Do they live near you or far away?
  - How often did you see each other?
- Do you speak to family and friends face to face/online?
- Are you seeing or speaking to teachers and students as part of school online?

- Are you involved in any online groups outside school – please describe (examples such as online choir, dancing classes, , youth groups/ youth theatre, Scouts/Guides, social activities etc)
- Do you have people who help you, such as teachers, Scout leaders, parents, grandparents, family friends? How did they help you, for example listening, help with homework, help with mental health?
- Can you tell us about any ways your friendship groups influence you such as encouraging you to get involved in things? Do you find you compare your life and theirs?
- Social roles possibly as a young carer

For peer review only - http://bmjopen.bmj.com/site/about/guidelines.xhtml

MENTAL HEALTH

How do you feel about the changes that have been brought about by Covid-19?
Have they had any impact on your mental health or wellbeing? Please tell us about these

- What are the things most bothering you at the moment?
- Have you experienced any impact on positive emotions? (prompts: concentration, being able to do and achieve things, relationships with others, how well you are managing and feelings of control over your situation?)
- Have you experienced any negative feelings? (prompts: such as, not enjoying things as much, anxiety, worry)
- Could you tell us if you have had any physical symptoms that might be due to being stressed or anxious? (prompts: fatigue, sleep problems, pain, illness symptoms, heart racing)

Have you been doing/ planning anything to help with this?

- Connecting with family or friends online
- Online groups?
- Hobbies/ Reading
- Exercise at home/ outside the home
- Volunteering
- Anything else you can tell me about?

Why are you doing/ not doing these things?

- Helpful/ not helpful – please tell us why
- Enjoyable
- Good for mental health/ wellbeing
- Can't get online, not connected, not comfortable, affordability, confidence in using/ skills
- Skills in using the internet/ communication software
- Living arrangements/ Work/ caring demands
- Peer support/ pressure
- Difficulties/ restriction in physical environment

PROSPECTION

Has the pandemic meant that you have any worries for the future?

How are these different from the worries you had before?

- Sense of control/ powerlessness
- Severity of worries / perspective

Will this change the way you live your life in future?

- The way you connect with others
- How you look after yourself
- How you support others
-

Has this changed what things are important to you?

For peer review only - http://bmjopen.bmj.com/site/about/guidelines.xhtml

Standards for Reporting Qualitative Research (SRQR)*

http://www.equator-network.org/reporting-guidelines/srqr/

Title and abstract

	Page/line no(s).
**Title** - Concise description of the nature and topic of the study Identifying the study as qualitative or indicating the approach (e.g., ethnography, grounded theory) or data collection methods (e.g., interview, focus group) is recommended	1, 1-3
**Abstract** - Summary of key elements of the study using the abstract format of the intended publication; typically includes background, purpose, methods, results, and conclusions	2, 1-17

Introduction

**Problem formulation** - Description and significance of the problem/phenomenon studied; review of relevant theory and empirical work; problem statement	3, 16-30
**Purpose or research question** - Purpose of the study and specific objectives or questions	4, 1-3

Methods

**Qualitative approach and research paradigm** - Qualitative approach (e.g., ethnography, grounded theory, case study, phenomenology, narrative research) and guiding theory if appropriate; identifying the research paradigm (e.g., postpositivist, constructivist/ interpretivist) is also recommended; rationale**	4, 5-6<br>5, 18-19
**Researcher characteristics and reflexivity** - Researchers' characteristics that may influence the research, including personal attributes, qualifications/experience, relationship with participants, assumptions, and/or presuppositions; potential or actual interaction between researchers' characteristics and the research questions, approach, methods, results, and/or transferability	4, 28-32
**Context** - Setting/site and salient contextual factors; rationale**	4, 28
**Sampling strategy** - How and why research participants, documents, or events were selected; criteria for deciding when no further sampling was necessary (e.g., sampling saturation); rationale**	4, 18-24
**Ethical issues pertaining to human subjects** - Documentation of approval by an appropriate ethics review board and participant consent, or explanation for lack thereof; other confidentiality and data security issues	4, 9-10<br>4, 14-16
**Data collection methods** - Types of data collected; details of data collection procedures including (as appropriate) start and stop dates of data collection and analysis, iterative process, triangulation of sources/methods, and modification of procedures in response to evolving study findings; rationale**	4, 29 (types of data)<br>5, 3-5 (responding to evolving findings)

For peer review only - http://bmjopen.bmj.com/site/about/guidelines.xhtml

	5, 27 – 6, 2 (iterative theme generation)<br>6, 4 (dates)
**Data collection instruments and technologies** - Description of instruments (e.g., interview guides, questionnaires) and devices (e.g., audio recorders) used for data collection; if/how the instrument(s) changed over the course of the study	Topic Guide in Supplementary Data
**Units of study** - Number and relevant characteristics of participants, documents, or events included in the study; level of participation (could be reported in results)	Table on p6, 11-13
**Data processing** - Methods for processing data prior to and during analysis, including transcription, data entry, data management and security, verification of data integrity, data coding, and anonymization/de-identification of excerpts	5, 17-27
**Data analysis** - Process by which inferences, themes, etc., were identified and developed, including the researchers involved in data analysis; usually references a specific paradigm or approach; rationale**	5, 17-27
**Techniques to enhance trustworthiness** - Techniques to enhance trustworthiness and credibility of data analysis (e.g., member checking, audit trail, triangulation); rationale**	5, 20

Results/findings

**Synthesis and interpretation** - Main findings (e.g., interpretations, inferences, and themes); might include development of a theory or model, or integration with prior research or theory	p6-16
**Links to empirical data** - Evidence (e.g., quotes, field notes, text excerpts, photographs) to substantiate analytic findings	p6-16

Discussion

**Integration with prior work, implications, transferability, and contribution(s) to the field** - Short summary of main findings; explanation of how findings and conclusions connect to, support, elaborate on, or challenge conclusions of earlier scholarship; discussion of scope of application/generalizability; identification of unique contribution(s) to scholarship in a discipline or field	17-19
**Limitations** - Trustworthiness and limitations of findings	19, 12-23

Other

**Conflicts of interest** - Potential sources of influence or perceived influence on study conduct and conclusions; how these were managed	1, 16-17
**Funding** - Sources of funding and other support; role of funders in data collection, interpretation, and reporting	1, 18-23

For peer review only

For peer review only - http://bmjopen.bmj.com/site/about/guidelines.xhtml

*The authors created the SRQR by searching the literature to identify guidelines, reporting standards, and critical appraisal criteria for qualitative research; reviewing the reference lists of retrieved sources; and contacting experts to gain feedback. The SRQR aims to improve the transparency of all aspects of qualitative research by providing clear standards for reporting qualitative research.

**The rationale should briefly discuss the justification for choosing that theory, approach, method, or technique rather than other options available, the assumptions and limitations implicit in those choices, and how those choices influence study conclusions and transferability. As appropriate, the rationale for several items might be discussed together.

Reference:

O'Brien BC, Harris IB, Beckman TJ, Reed DA, Cook DA. **Standards for reporting qualitative research: a synthesis of recommendations.** *Academic Medicine*, Vol. 89, No. 9 / Sept 2014 DOI: 10.1097/ACM.0000000000000388

For peer review only

For peer review only - http://bmjopen.bmj.com/site/about/guidelines.xhtml
